# Plastid Genome Evolution Across the Roridulaceae–Sarraceniaceae Clade (Ericales) in Relation to Carnivorous Strategies

**DOI:** 10.1002/ece3.73333

**Published:** 2026-03-26

**Authors:** Shengxin Chang, Peng Wang, Wei Han, Baiyin Yu, Chunxia Li

**Affiliations:** ^1^ College of Biology and Agriculture/Guangdong Provincial Key Laboratory of Utilization and Conservation of Food and Medicinal Resources in Northern Region Shaoguan University Shaoguan Guangdong China; ^2^ Institute of Tropical Crop Genetic Resources Chinese Academy of Tropical Agricultural Sciences Haikou Hainan China

**Keywords:** carnivory strategies, plastome evolution, RNA editing, Roridulaceae–Sarraceniaceae clade

## Abstract

The Roridulaceae–Sarraceniaceae (RS) clade within Ericales exhibits strikingly divergent carnivorous strategies. To investigate how these differences shape plastid genome (plastome) evolution, we analyzed four representative species: *Roridula gorgonias* (Roridulaceae), which absorbs nutrients from insect feces without digestive enzymes; and three pitfall trap Sarraceniaceae species—*Heliamphora minor* (relying on microbial decomposition), *Darlingtonia californica* (with digestive enzymes, lacking endocytosis), and *Sarracenia leucophylla* (with digestive enzymes and endocytosis). Across RS species, *ndh* genes frequently exhibit severely disrupted or completely lost open reading frames (ORFs). Compared with Sarraceniaceae species exhibiting greater degrees of carnivorous specialization, *Roridula gorgonias* plastomes exhibit markedly accelerated and structurally dynamic evolution, including partial loss of the *clpP* gene, frequent insertion events, elevated nonsynonymous (*d*_N_) and synonymous (*d*_S_) substitution rates, widespread relaxation of purifying selection, multiple sites under positive selection across plastome genes, and reduced RNA editing efficiency at conserved codons. In contrast, variation in digestive traits and endocytotic capability among Sarraceniaceae species corresponds to only subtle plastome‐level differences, with limited changes in GC content, gene number, and evolutionary rates. These results indicate that plastome evolution within the RS clade is not predicted by the degree of carnivorous specialization alone, but instead is best explained by context‐dependent effects of trophic strategy and other ecological pressures. In *Roridula gorgonias*, the pronounced plastome dynamics likely reflect the combined influence of carnivory and recurrent wildfire stress in nutrient‐poor habitats, thereby facilitating the accumulation of higher mutational loads.

## Graphical abstract

The Roridulaceae–Sarraceniaceae clade exhibits strikingly divergent carnivorous strategies, providing a framework to examine how trophic shifts influence plastid genome evolution. Comparative plastome analyses reveal recurrent *ndh* gene disruption across species, while *Roridula gorgonias* shows pronounced genomic restructuring, including elevated substitution rates, widespread relaxation of selection, and reduced RNA editing efficiency. These findings indicate that plastome evolution within the RS clade is not predicted solely by carnivorous specialization, but instead reflects context–dependent interactions between trophic strategy and ecological pressures such as recurrent wildfire disturbance.

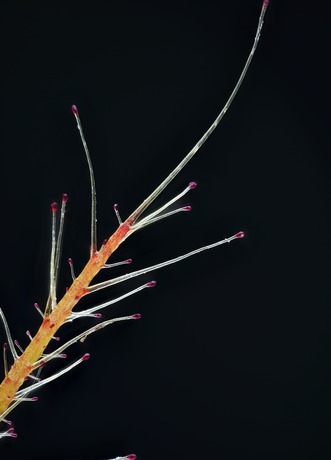

## 1 Introduction

To cope with nutrient‐poor environments, some higher plants have evolved carnivorous strategies, which lead to lower mass‐based photosynthetic rates, reduced photosynthetic nutrient‐use efficiency, and lower allocation of nitrogen to photosynthesis (Ellison 2006; Pavlovič and Saganová 2015). At the plastome level, this shift in nutritional strategy has been associated with distinct evolutionary changes relative to obligate autotrophs. For example, plastomes of carnivorous species in Lentibulariaceae and Droseraceae commonly exhibit loss or pseudogenization of specific photosynthetic genes (Wicke et al. 2014; Nevill et al. 2019). Within the carnivorous Lentibulariaceae family, the substitution rate and microstructural changes across the entire plastome significantly increase, with a notable rise in nonsynonymous substitutions in protein‐coding genes (Wicke et al. 2014). In the carnivorous Droseraceae family, the plastomes undergo multiple rearrangements and exhibit substantial variation of the inverted repeat (IR) region (Nevill et al. 2019). Despite these insights, the evolutionary patterns of the plastome during the transition of plants from obligate autotrophy to carnivory remain poorly understood.

Currently, plants with carnivorous capabilities are classified into two distinct groups: proto‐carnivorous plants, which can capture small animals but lack the ability to digest them directly, and full‐fledged carnivorous plants, which produce digestive enzymes to break down their prey (Ellison and Adamec 2018). Taxonomically, most orders contain either one type or the other. For instance, genera within the Poales order, such as Paepalanthus, Catopsis, and Brocchinia, are considered proto‐carnivorous as they lack digestive enzymes (Givnish et al. 1984; Adlassnig et al. 2011). In contrast, orders like Alismatales, Oxalidales, Nepenthales, and Lamiales exclusively comprise full‐fledged carnivorous plants (Pereira et al. 2012; Sirová et al. 2003; Kocáb et al. 2020; Płachno et al. 2024; Fukushima et al. 2017). A notable exception to this taxonomic pattern is the RS clade within the Ericales, which contains both types of carnivorous plants.

All species belonging to the Sarraceniaceae family (*Sarracenia* L., *Darlingtonia* Torr., and *Heliamphora* Benth.) are equipped with pitfall traps and cuticular pores that facilitate them in capturing and absorbing nutrients from their prey. Among the *Sarracenia* species, like *Sarracenia leucophylla* and *Sarracenia psittacina* , secrete proteases for digestion, while others, such as *Sarracenia rosea* and *Sarracenia purpurea ssp. purpurea* , rely on microbial assistance (Koller‐Peroutka et al. 2019; Ellison and Adamec 2018). *Darlingtonia californica* , the sole species in its genus, was initially believed to lack digestive enzymes (Ellison and Farnsworth 2005). However, recent rigorous tests have confirmed its ability to produce digestive enzymes by adding tetracycline to prevent exogenous bacterial proteases from falsifying the results (Koller‐Peroutka et al. 2019). Within the *Heliamphora* genus, only *Heliamphora tatei* exhibited proteolytic activity, whereas species like *Heliamphora minor* and *Heliamphora heterodoxa* did not, relying instead on the enzymes of symbiotic bacteria to break down their prey (Jaffe et al. 1992; Ellison and Adamec 2018). Furthermore, some *Sarracenia* species exhibit endocytosis, a mechanism suggested to represent a more advanced stage of nutrient uptake in carnivorous plants, enabling the absorption of whole proteins or large fractions thereof (Ellison and Adamec 2018; Freund et al. 2022). However, this capability currently is not found in either *Darlingtonia* or *Heliamphora* genera (Koller‐Peroutka et al. 2019).

Intriguingly, the Roridulaceae family, comprising only the genus *Roridula*, employs flypaper traps to capture small animals instead of pitfall traps. Despite possessing cuticular pores similar to Sarraceniaceae, Roridulaceae plants lack digestive enzymes. Instead, they attract carnivorous animals by capturing small insects and absorbing the excreta left on their leaves (Givnish 2015). This adaptive strategy positions *Roridula* as a crucial transitional form—and likely the most primitive extant lineage—between obligate autotrophic plants and full‐fledged carnivorous plants (Ellison and Adamec 2018). Notably, fossil evidence dating back 35–47 million years ago confirms the primitive nature of this carnivorous mechanism (Givnish 2015). The “spectrum” of carnivorous strategies within the RS families provides a window into the plastome evolution of carnivorous plants.

In addition to carnivorous plants, the Ericales order also comprises obligate heterotrophic plants, including holoparasitic plants within the Mitrastemonaceae family and mycoheterotrophic plants within the Ericaceae family. Remarkably, the plastomes of these nutritionally diverse plants have been publicly available (Shyu 2013; Braukmann et al. 2017), and the plastomes of the main autotrophic lineages within Ericales have also been sequenced (Rose et al. 2018; Yan et al. 2018). These plastome information provides a robust foundation for the plastome evolution studying.

Given this, our present study focused on four RS plants that exhibit a gradient in their carnivorous capabilities: *Roridula gorgonias* (flypaper trap without digestive enzymes and not deriving nutrients from prey directly) (Givnish 2015), *Heliamphora minor* (pitfall trap lacking digestive enzymes but using microorganisms to decompose prey) (Jaffe et al. 1992), *Darlingtonia californica* (pitfall trap with digestive enzymes but no endocytosis) (Koller‐Peroutka et al. 2019), and *Sarracenia leucophylla* (pitfall trap with both digestive enzymes and endocytosis) (Koller‐Peroutka et al. 2019). These species span key transitional stages along the carnivory continuum, from the primitive *Roridula gorgonias* to the highly specialized *Sarracenia leucophylla* . This gradient provides an ideal framework for exploring how increasing dependence on carnivorous nutrient acquisition correlates with structural and functional changes in the plastome.

To investigate this, we sequenced the plastomes and transcriptomes of these plants and conducted a comprehensive comparative analysis in conjunction with the plastomes of other nutritional types within the Ericales order (Table S1), aiming to explore clues to the evolution of the plastome during the transition of plants from obligate autotrophy to carnivory.

## 2 Materials and Methods

### 2.1 Selected Species and Genomes

The species selected for this study include four RS carnivorous plants: *Roridula gorgonias*, *Heliamphora minor*, *Darlingtonia californica* , and *Sarracenia leucophylla* . In addition to these, the comparative plastome study involves species from key taxonomic units within Ericales. The autotrophic species examined are: *Barringtonia racemosa* (NC_035705.1), *Pterostyrax hispidus* (NC_041135.1), *Saurauia tristyla* (NC_044098.1), *Sladenia celastrifolia* (NC_035707.1), *Diospyros hainanensis* (NC_042160.1), *Marcgravia coriacea* (NC_041255.1), *Fouquieria diguetii* (MG524997.1), *Pouteria campechiana* (KX426215.1), *Euryodendron excelsum* (NC_039178.1), *Clethra fargesii* (NC_060326.1), *Symplocos ovatilobata* (NC_036489.1), *Aegiceras corniculatum* (MN167882.1), *Apterosperma oblate* (NC_035641.1), *Polemonium chinense* (NC_050355.1), and *Hydrocera triflora* (NC_037400.1). The obligate heterotrophic species include: *Allotropa virgate* (NC_035580.1), *Pityopus californicus* (NC_035584.1), *Hemitomes congestum* (NC_035581.1), *Monotropa uniflora* (NC_035582.1), *Monotropsis odorata* (NC_035583.1), and *Mitrastemon kanehirai* (MF372930.1). The *Nicotiana tabacum* plastome (NC_001879) was used as the reference genome for gene annotation, genome comparison, and evolutionary rate analysis, owing to its extensive characterization and well‐established plastid gene functions (Narra et al. 2025). For the comparative transcriptome analysis, autotrophic models included *Nicotiana tabacum* (SRR11674778) and *Solanum lycopersicum* (SRR8749284).

### 2.2 Genome Sequencing, Assembly, and Annotation

The seedlings of the four RS carnivorous plant species were grown in a controlled greenhouse under a 12‐h light/12‐h dark photoperiod, at temperatures ranging from 20°C to 30°C and a constant relative humidity of 80%. Total genomic DNA was individually extracted from fresh plant material using the CTAB method. All samples were treated with RNAse (Qiagen) before purification by PEG‐8000 precipitation. High‐molecular weight DNA was subjected to whole genome shotgun sequencing with an Illumina NovaSeq sequencing platform, which generated 40.4–41.8 million 2 × 150 bp reads for the samples. The raw reads were trimmed for adapters and low‐quality bases using fastp v0.23.1 (Chen et al. 2018) with the following parameters: ‐l 50, ‐‐qualified_quality_phred 20, ‐‐n_base_limit 5, ‐‐unqualified_percent_limit 40, ‐‐trim_poly_g, and ‐‐trim_poly_x, producing high‐quality paired‐end reads. The trimmed reads were then assembled into plastomes using NOVOPlasty v.4.3.1 (Dierckxsens et al. 2017) with a chloroplast‐type configuration and an appropriate seed sequence. The reads were then mapped to the assembled sequences using Bowtie2 v.2.3.5 (Langmead and Salzberg 2012), and the connections of IR regions and single copy regions were verified manually.

The plastome of tobacco and known plastomes of Ericales were utilized as references for the initial genome annotation, employing the Live Annotation & Predict feature in Geneious Prime v.2023.0.1 (www.geneious.com) with a similarity setting of 60%. Manual curation of ORF annotations was performed using the coding sequences (CDS) of obligate autotrophic plants as references. For protein‐coding genes, the annotated ORFs were aligned to the tobacco CDS using Bio.Align.PairwiseAligner (https://biopython.org/docs/dev/Tutorial/chapter_pairwise.html) in Python, with coverage calculated in global alignment mode (match: 1, mismatch: ‐1, gap penalties: ‐5/‐1). ORFs with ≥ 70% coverage to tobacco CDS were predicted as intact genes, while those with < 70% coverage were predicted as pseudogenes or missing. Additionally, for *ndh* genes, if any *ndh* gene is annotated as a pseudogene or is lost, all other *ndh* genes will also be annotated as functionally lost, regardless of whether they have a conserved ORF (as detailed in the discussion section). tRNA genes were annotated using tRNAscan‐SE v2.0 (Chan et al. 2021) with organelle mode (‐O) and intron detection (‐I), and genes containing introns were manually verified.

### 2.3 Transcriptome Sequencing and Analysis

Total RNA was isolated from fresh leaves of the four RS carnivorous species using Trizol reagent (Life Technologies Corporation, USA). Strand‐specific RNA‐seq reads were then generated using the Illumina NovaSeq sequencing platform, following treatment with DNase I (Invitrogen, USA), rRNA depletion using the Illumina Ribo‐Zero rRNA Removal Kit (Plant Leaf version) (Illumina, USA), and cDNA library construction with dUTP and random hexamers. Trim Galore v0.6.8 (https://github.com/FelixKrueger/TrimGalore) was used to trim adapters and low‐quality bases (minimum Phred score = 20, minimum read length = 50 bp, adapter stringency = 3), producing high‐quality paired‐end reads.

The trimmed reads were mapped to their corresponding plastomes using TOPHAT2 (Kim et al. 2013) with relaxed parameters (‐‐library‐type fr‐firststrand ‐‐read‐mismatches 4 ‐‐read‐gap‐length 0 ‐‐read‐edit‐dist 4 ‐‐maxinsertion‐length 0 ‐‐max‐deletion‐length 0 ‐‐coverage‐search). The reads for designated regions on each strand were counted using Geneious Prime. FPKM values were calculated as follows: (Fragment number for designated feature × 10^6^)/(Total fragment number in sample × Feature length in kilobases), where “Total fragment number in sample” represents the total number of fragments mapped to the positive and negative strands of the plastome.

Differential gene expression between carnivorous plants and obligate autotrophic model plants was analyzed using DESeq2 v.3.22 (Love et al. 2014). Genes with −log_10_(*p*adj) > 1 were considered statistically significant, with padj representing FDR‐adjusted *p*‐values calculated using the Benjamini–Hochberg procedure. The “Find Variations” function in Geneious Prime was utilized to search for RNA editing sites on the protein‐coding transcripts, using the following thresholds: minimum coverage = 60, minimum variant frequency = 10%, maximum variant *p*‐value = 10^−6^, and minimum strand‐bias *p*‐value = 10^−5^ when exceeding 65% bias.

### 2.4 Genome Repeat and Mismatch Region Analysis

The plastome with a single IR copy was self‐aligned using BLASTN v.2.12.0 (Camacho et al. 2009) to search for the short repeats with a word size of 7. Repeats shorter than 500 bp were considered short fragment repeats. Repeats in different regions of the genome were filtered and merged using Bedtools v.2.30.0 (Quinlan and Hall 2010). The genome alignment map was generated using the progressiveMauve algorithm implemented in Mauve v2.4.0 (Darling et al. 2004) with default parameters. For mismatch region analysis, the plastome with a single IR copy were aligned using BLASTN with a word size of 7. The low‐homologous regions in multiple genomes were identified using bedtools complement. The mismatched regions were aligned against the NCBI nt/nr database (https://www.ncbi.nlm.nih.gov/) using BLASTN, with an *E*‐value threshold of < 1e−5, to infer their potential source.

### 2.5 Evolutionary Rate Estimation

MAFFT v.7.511 (Katoh and Standley 2013) was used to align CDS sequences in codon mode. The alignment results were optimized using MACSE v.2.06 (Ranwez et al. 2018) with default parameters. Subsequently, the sequences were cleaned using Gblocks v.0.91b (Castresana 2000) in codon mode. The parameter settings included minimum length of a block = 5, maximum number of contiguous nonconserved positions = 8, allowed gap position = none. The cleaned gene sequences were concatenated end‐to‐end, and a phylogenetic tree was constructed based on the study by Rose et al. (2018). ModelFinder in IQ‐TREE v.2.0.7 (Minh et al. 2020) was used to select DNA substitution models, with criterion = BIC. All of the above steps were implemented in PhyloSuite v1.2.3. (Zhang et al. 2020). Finally, the MG94 × GTR substitution model similar to the optimal model was selected for maximum likelihood analysis using Hyphy v.2.2.4 (Kosakovsky Pond et al. 2020). This model allowed for the estimation and optimization of branch lengths on the phylogenetic tree. CODEML from PAML v4.10.9 (Yang 2007) was used to analyze base substitution rates in CDS region of protein‐coding genes, with parameters including runmode = pairwise and codonFreq = F3*4. For non‐CDS regions, base substitution rate analysis was conducted using BASEML with the tree and model = HKY85.

Codon‐based selection analyses were conducted using ETE3 v3.1.3 (https://pypi.org/project/ete3/) interfaced with CODEML. For each gene, obligate autotrophic Ericales species (Table S1) were designated as background branches, whereas heterotrophic lineages were treated as foreground branches. Branch models included a one‐ratio model across the tree (M0), a model with the foreground branch fixed at neutrality (b_neut), and a model allowing the foreground branch to have an independent ω (b_free). Genes with a significant difference between b_free and M0 (LRT, *p*‐value < 0.05) were further classified by “Constraint_status,” with “Relaxed” when foreground ω exceeded background ω, or “Strengthened” when foreground ω was lower than background ω. The relative change in selective pressure was summarized as Δω_fg/bg = (ω_fg − ω_bg)/ω_bg. Genes were considered under relaxed selection if b_free differed significantly from M0 (*p* value < 0.05) but the foreground ω did not significantly deviate from neutrality in comparison to b_neut (*p* value ≥ 0.05). Branch‐site models (bsA1 vs. bsA) were used to detect site‐specific positive selection along foreground branches (LRT, *p* value < 0.05). Positively selected sites were identified using Bayes Empirical Bayes (BEB) with posterior probability ≥ 0.95, and summarized as the BEB site ratio (number of selected sites/protein length). All LRTs were calculated as 2Δℓ = 2(ℓ₁ − ℓ₀) with degrees of freedom equal to the difference in free parameters. Genes with extreme ω estimates (ω ≥ 10 or ω ≤ 0.001 in any model) were excluded from downstream analyses to avoid potential artifacts.

### 2.6 Statistical Analysis

All statistical analyses were performed in R (https://www.r‐project.org/). For two‐group comparisons, the Mann–Whitney test was used for significance testing, and group differences were quantified using effect size estimates with 95% confidence intervals. For multiple‐group comparisons, one‐way ANOVA followed by LSD post hoc test was used to identify significant differences. Normality of each group was evaluated using the Shapiro–Wilk test, and homogeneity of variance was assessed with Levene's test. For pairwise correlation analyses, Pearson correlation was applied when both variables were approximately normally distributed, while Spearman correlation was used when normality assumptions were not met. *p* values < 0.05 were considered significant for group comparisons, and *p* values < 0.01 were considered significant for correlation analyses.

## 3 Results

### 3.1 Genome Size, Gene Content, and Nucleotide Composition

**FIGURE 1:**
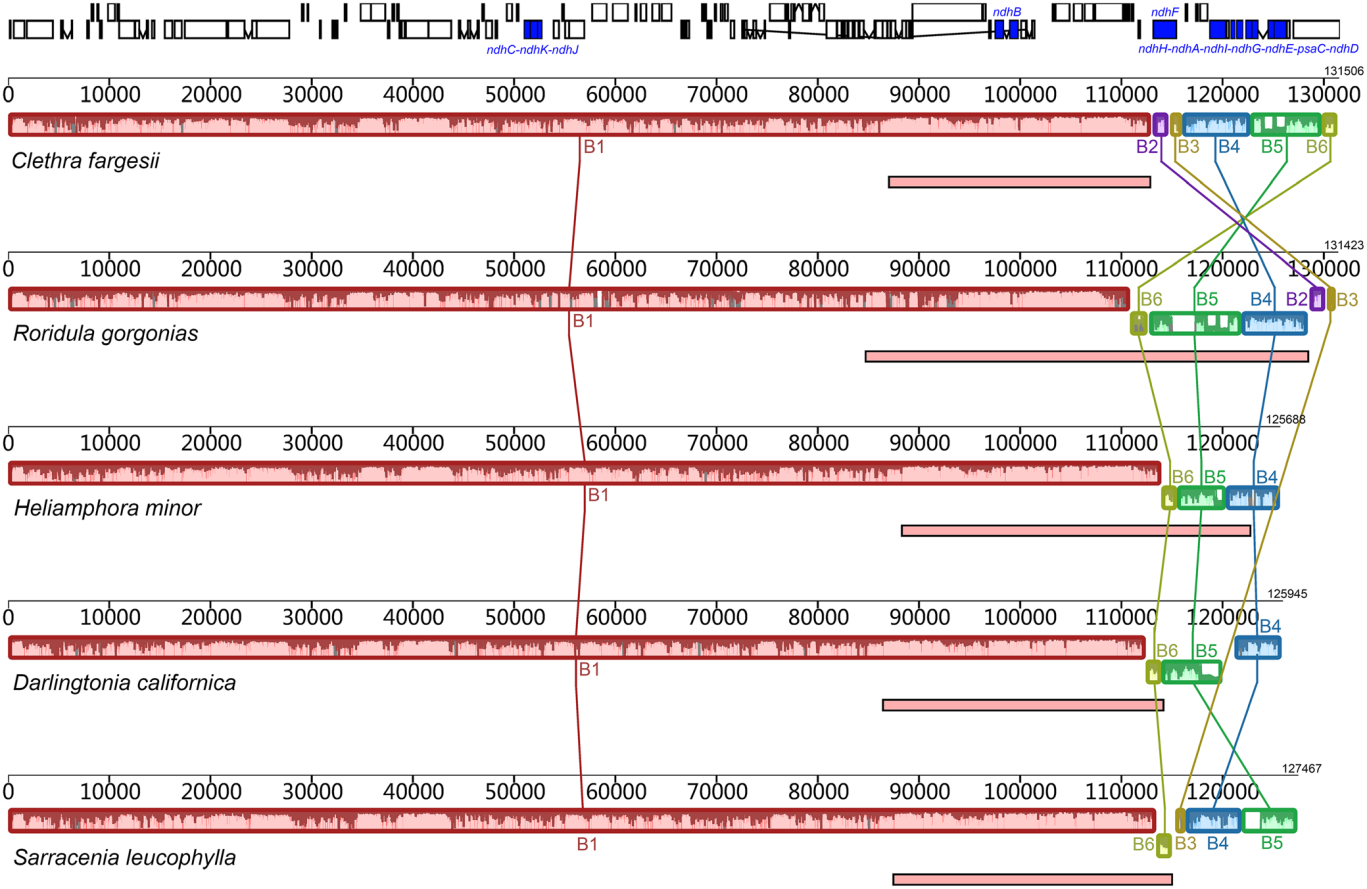
Comparison of plastomes between RS carnivorous plants and a closely related autotrophic plant (*Clethra fargesii*). The second IR copy was excluded from each plastome prior to alignment. The locally collinear blocks (LCBs) with same color indicate homologous regions. The lines within these blocks represent the degree of sequence similarity. The blue blocks in the annotation track at the top of the figure indicate the presence of *ndh* genes. The light red rectangles beneath each genome represent the IR region.

Within the RS clade, the plastome of *Roridula gorgonias* exhibits the longest non‐redundant length (with one IR retained), measuring 131.4 kb (Figure 1). This length is comparable to that of the representative obligate autotrophic plants in the Ericales order (Table S1). In contrast, the non‐redundant plastome lengths of the three Sarraceniaceae species are significantly shorter than those of obligate autotrophic plants, being on average 4.4% shorter (95% CI: 3.1%–4.9%), yet substantially longer than those of obligate heterotrophic plants, on average 2.6 times longer (95% CI: 2.0–3.2).

**FIGURE 2:**
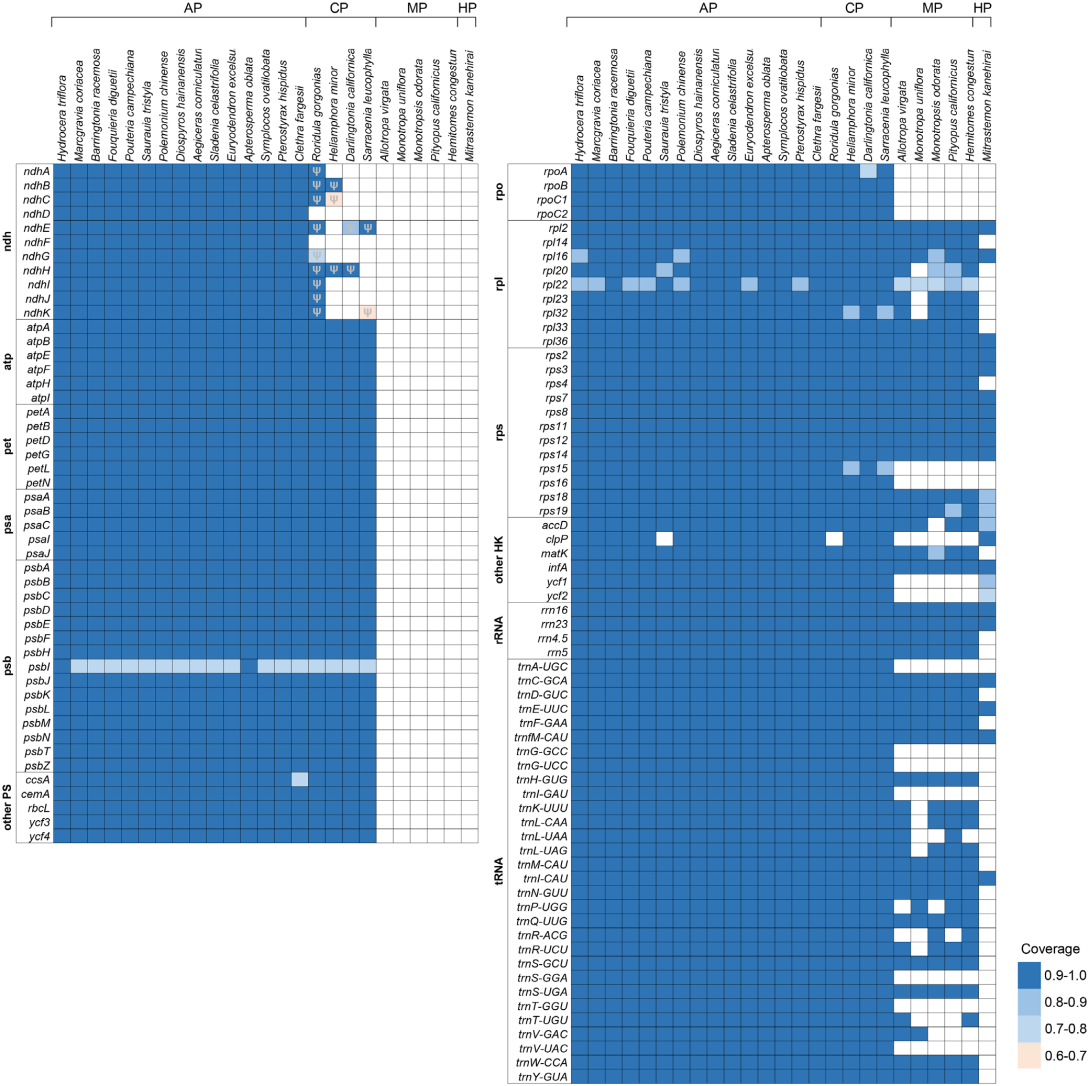
Distribution of plastid genes across Ericales plants. The filled blocks indicate the presence of ORFs with at least 60% coverage compared to the tobacco CDS, represented by a heatmap. Regions with coverage below 60% have no color fill. Coverage is defined as the proportion of non‐gap bases in the tobacco sequence that are aligned with corresponding bases in the query sequence. The symbol ψ denotes pseudogenes. PS and HK denote photosynthetic genes and housekeeping genes, respectively. AP, CP, MP, and HP denote obligate autotrophic, carnivorous, mycoheterotrophic, and holoparasitic plants, respectively.

Compared with the plastid CDS of obligate autotrophic plants, numerous *ndh* gene ORFs in the four carnivorous species have undergone shortening (Figure 2). In the typical plastome of autotrophic plants, *ndhB* and *ndhF* exist as independent genes, while the remaining *ndh* genes are organized into two distinct clusters: *ndhJ–ndhK–ndhC* and *ndhH–ndhA–ndhI–ndhG–ndhE–psaC–ndhD* (Figure 1). In *Roridula gorgonias*, the ORFs of *ndhB* and the *ndhJ–ndhK–ndhC* cluster remain intact. By contrast, in the three Sarraceniaceae species, these two loci have experienced premature ORF terminations, although the lengths of homologous regions are largely preserved (Figure S1).

**FIGURE 3:**
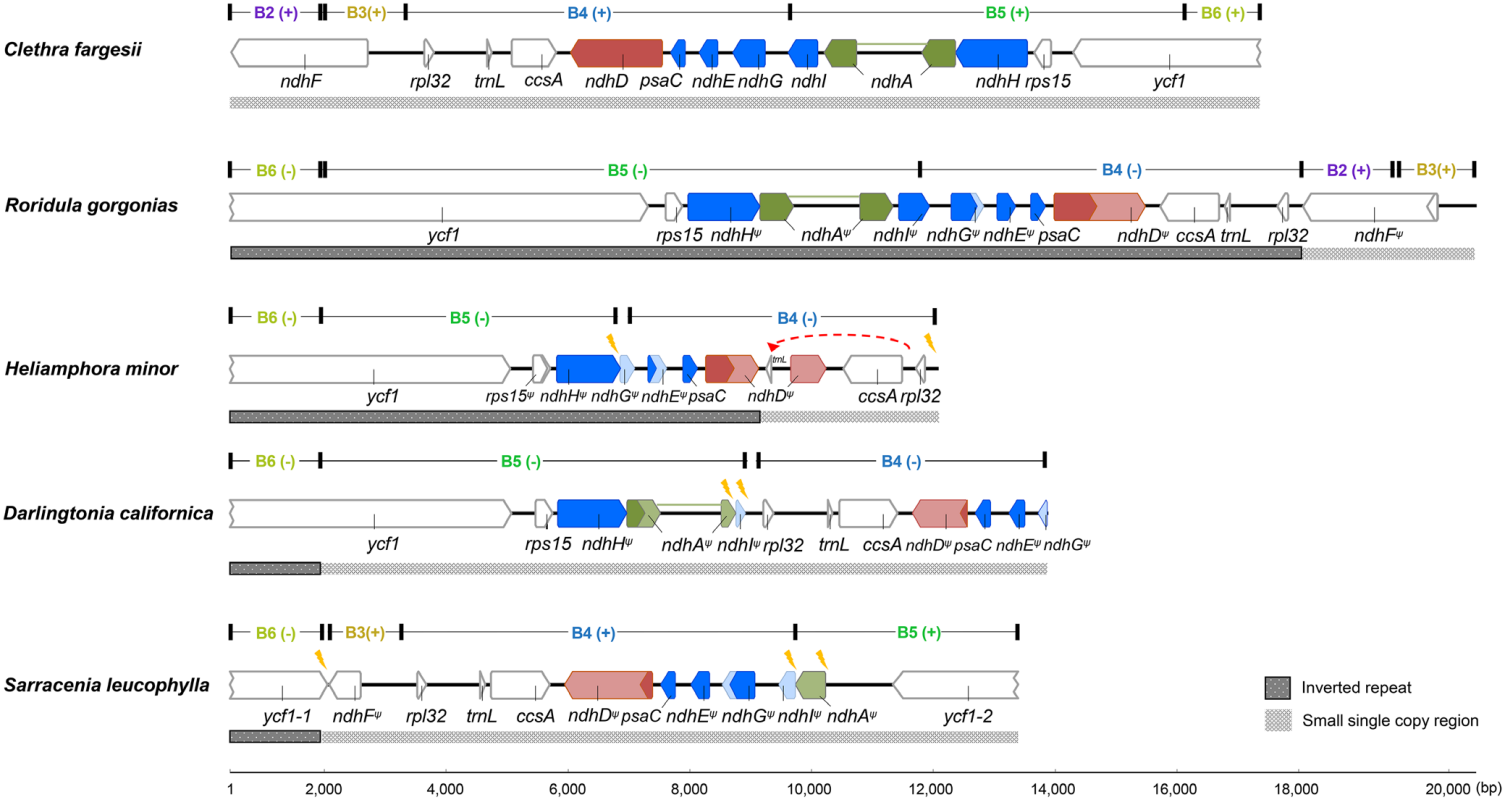
DNA rearrangement within the small single copy (SSC) region of plastomes in RS carnivorous plants. Colored arrows represent the *ndhH–ndhA–ndhI–ndhG–ndhE–psaC–ndhD* cluster, while arrows without color indicate flanking genes. Dark‐colored arrow regions denote ORFs, and the light‐colored arrows represent the areas homologous to gene sequences in autotrophic plants. The direction of the arrows indicates the orientation of the genes. Lightning bolt symbols mark DNA deletions. The symbol ψ denotes pseudogenes. The labels B2–B5 correspond to the mauve LCBs in Figure 1, where the orientation of each LCB is annotated in parentheses: “+” indicates the same orientation as in *Clethra fargesii*, while “−” indicates the reverse orientation relative to *Clethra fargesii*.

Genetic variations in *ndhF* and the *ndhH–ndhA–ndhI–ndhG–ndhE–psaC–ndhD* cluster have been observed in both Roridulaceae and Sarraceniaceae (Figure 1). Specifically, in Roridulaceae, the ORFs of *ndhD* and *ndhF* are less than 50% of the length observed in autotrophic plants due to premature terminations. In Sarraceniaceae, variations mainly involve deletions of gene fragments (Figure 3). For example, *ndhA* and *ndhI* are completely absent in *Heliamphora minor*, partial sequences of *ndhA* are lost in *Darlingtonia californica* and *Sarracenia leucophylla* , and the entire *ndhF* gene is missing in both *Heliamphora minor* and *Darlingtonia californica* . These gene losses contribute substantially to the observed plastome shortening in Sarraceniaceae.

Beyond the *ndh* genes, a few housekeeping genes have experienced sporadic ORF shortening. The ORFs of *rpoA* in *Darlingtonia californica* , and *rpl32* and *rps15* in *Heliamphora minor* and *Sarracenia leucophylla* retain 79.0%–89.8% of the length of the conserved CDS in a reference autotrophic plant (tobacco) and were predicted as intact genes (Figure 2). The *psbI* gene in these carnivorous plants retains only 71% of the CDS length in tobacco; however, such variation is commonly observed among autotrophic plants in Ericales, and it was accordingly annotated as an intact gene (Figure 2). In contrast, *clpP* has lost exon 1, and the residual sequences of exons 2 and 3 exhibit only 65.7% identity to the CDS regions of obligate autotrophic plants; therefore, *clpP* has undergone functional loss.

**FIGURE 4:**
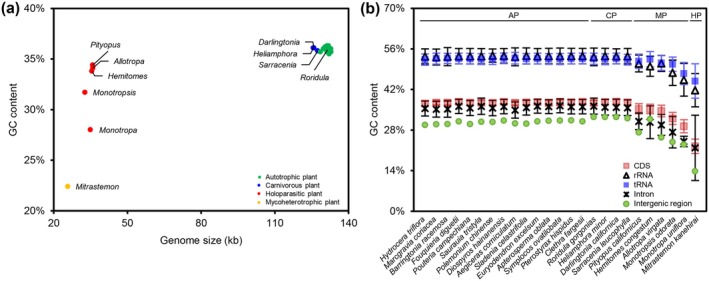
(a) Relationship between GC content and non‐redundant length of Ericales plastomes. The horizontal axis displays the plastome length excluding one IR copy. (b) GC content in different plastome regions, with error bars representing 95% confidence intervals for each species. AP, CP, MP, and HP denote obligate autotrophic, carnivorous, mycoheterotrophic, and holoparasitic plants, respectively.

The GC content of RS plastomes closely resembles that of obligate autotrophic plants, hovering around 36% (Figure 4a). On a broader scale, as we transition from obligate autotrophic plants to carnivorous plants, and then to obligate heterotrophic plants (mycoheterotrophic and holoparasitic plants) within the Ericales order, a clear downward trend emerges in both the length and GC content of the plastomes. This trend, observed across different nutritional types, is also reflected in terrestrial mycoheterotrophs and holoparasites with varying degrees of heterotrophy (Wicke and Naumann 2018). Furthermore, although the GC content of distinct functional regions within Ericales plastomes shows considerable variation, the downward trajectory of these regions remains consistent throughout the transition from autotrophy to heterotrophy (Figure 4b).

### 3.2 Large Inverted Repeats and Short Repeats

The average IR length in RS carnivorous plants is 33.4 ± 6.6 kb, significantly greater than in obligate autotrophic plants (on average 7.4 kb longer, 95% CI: 2.3–12.5 kb) and substantially exceeding the lengths observed in obligate heterotrophic plants, where the IR region is often notably reduced or even lost (Table S1). When compared to obligate autotrophic plants, the IR regions in RS carnivorous plants exhibit conservation at their junctions with the large single‐copy region. Specifically, the flanking genes at these junctions are *rps19‐rpl2* and *rpl2‐trnH*‐GUG, respectively (Figure S2). However, the junctions between the IR regions and the SSC region are highly variable. In *Roridula gorgonias*, the IR has incorporated nearly the entire SSC region, with the sole exception of the *ndhF* gene. In contrast, in *Heliamphora minor*, the IR has integrated segments of the SSC, specifically encompassing B4, B5, and B6 LCBs. In the remaining two Sarraceniaceae species, the IR has incorporated portions of B6 LCB that contain the *ycf1* fragment (Figure 3). These integration events constitute major factors contributing to the elongated IR regions observed in RS plastomes.

**FIGURE 5:**
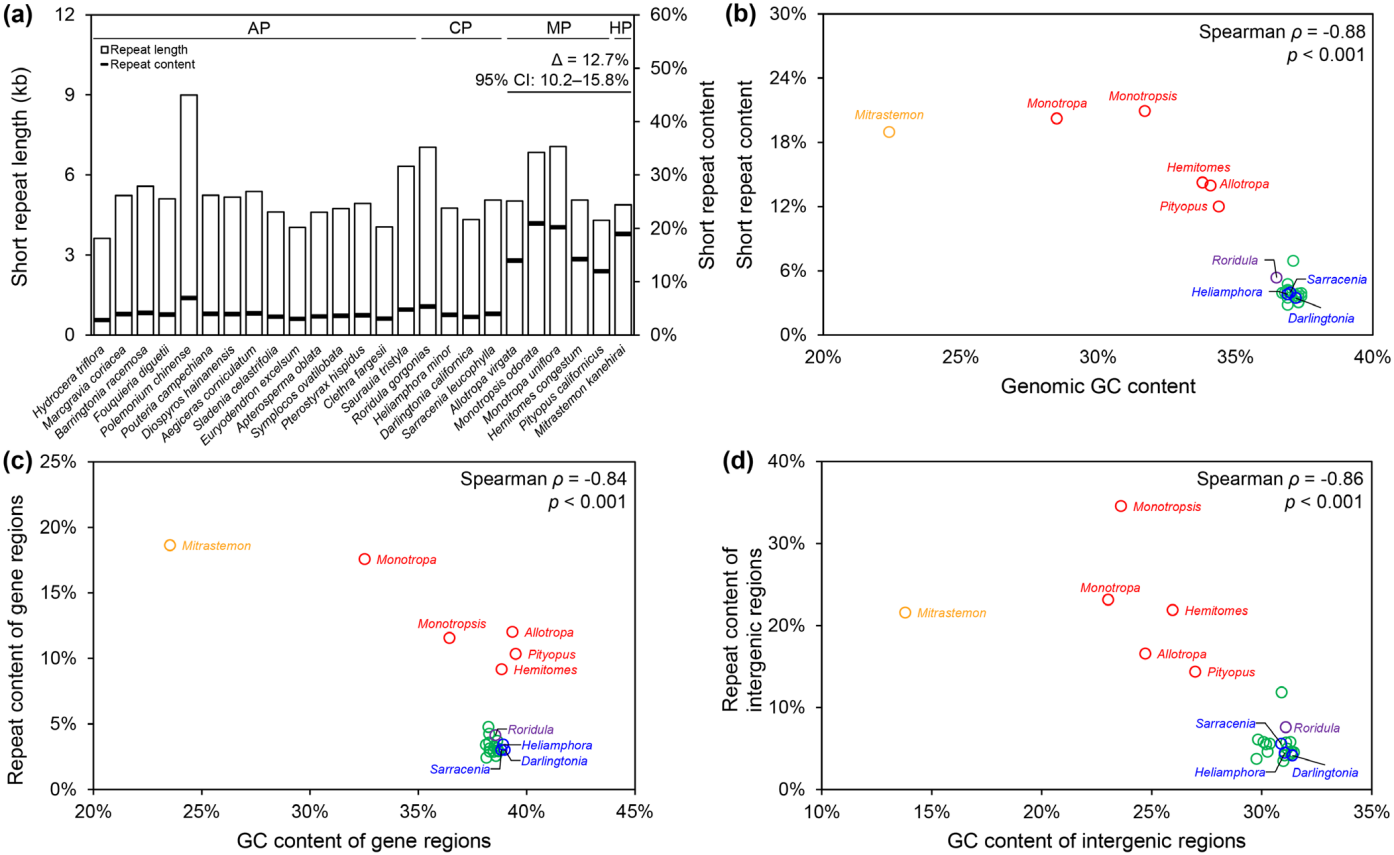
Short repeat content and correlations with GC content in Ericales plastomes. (a) Total length and content of short repeats. Differences in short repeat content between AP/CP and MP/HP are shown as effect size estimates with 95% confidence intervals, derived from the Mann–Whitney test. (b) Correlation between GC content and short repeat content. (c) Correlation between GC content and short repeat content in gene regions. (d) Correlation between GC content and short repeat content in intergenic regions. AP (green), CP (purple/blue), MP (red), and HP (orange) stand for obligate autotrophic, carnivorous, mycoheterotrophic, and holoparasitic plants, respectively.

To delve deeper into the plastomic landscapes of these plants, we employed the BLASTN algorithm to search for short repeats, varying the *E*‐value thresholds (Figure S3a). When comparing the slopes of the linear relationship between *E*‐value thresholds and short repeat content, carnivorous plants and obligate autotrophic plants showed significantly lower slopes than obligate heterotrophic plants (Figure S3b), suggesting they have a lower proportion of low‐similarity repeats. When applying a relaxed *E*‐value threshold of 6 to avoid the loss of low‐similarity repeats, we found that *Roridula gorgonias* had the highest content and length of short repeats among the RS species. The differences in short repeat content and length among the other three Sarraceniaceae species were not obvious (Figure 5a). Similar levels of variation in RS carnivorous plants were observed in obligate autotrophic plants. However, compared to obligate heterotrophic plants, both carnivorous and obligate autotrophic plants exhibited lower repeat content (Figure 5a). Furthermore, a comprehensive analysis at the whole‐plastome level revealed a significant negative correlation between short repeat content and GC content, which was consistently observed in both genic and intergenic regions (Figure 5b–d).

### 3.3 Homologous Recombination and Mismatch Regions

Compared to the plastomes of obligate autotrophic plants, the SSC region of RS carnivorous plants displays a series of rearrangement events (Figure 1). Major rearrangement events are concentrated in B2–B6 LCBs. In *Roridula gorgonias*, the B4–B6 LCBs are inserted in reverse orientation before B2–B3 LCBs, while in *Sarracenia leucophylla* , the B6 LCB is inserted in reverse orientation before B3–B5 LCBs. In *Heliamphora minor* and *Darlingtonia californica* , B4–B6 and B5–B6 LCBs occur reverse rearrangements, respectively. Additionally, a minor rearrangement occurs in B4 LCB of *Heliamphora minor*, where the *trnL*‐UAG gene undergoes reverse insertion into the *ndhH–ndhA–ndhI–ndhG–ndhE–psaC–ndhD* gene cluster (Figure 3).

**FIGURE 6:**
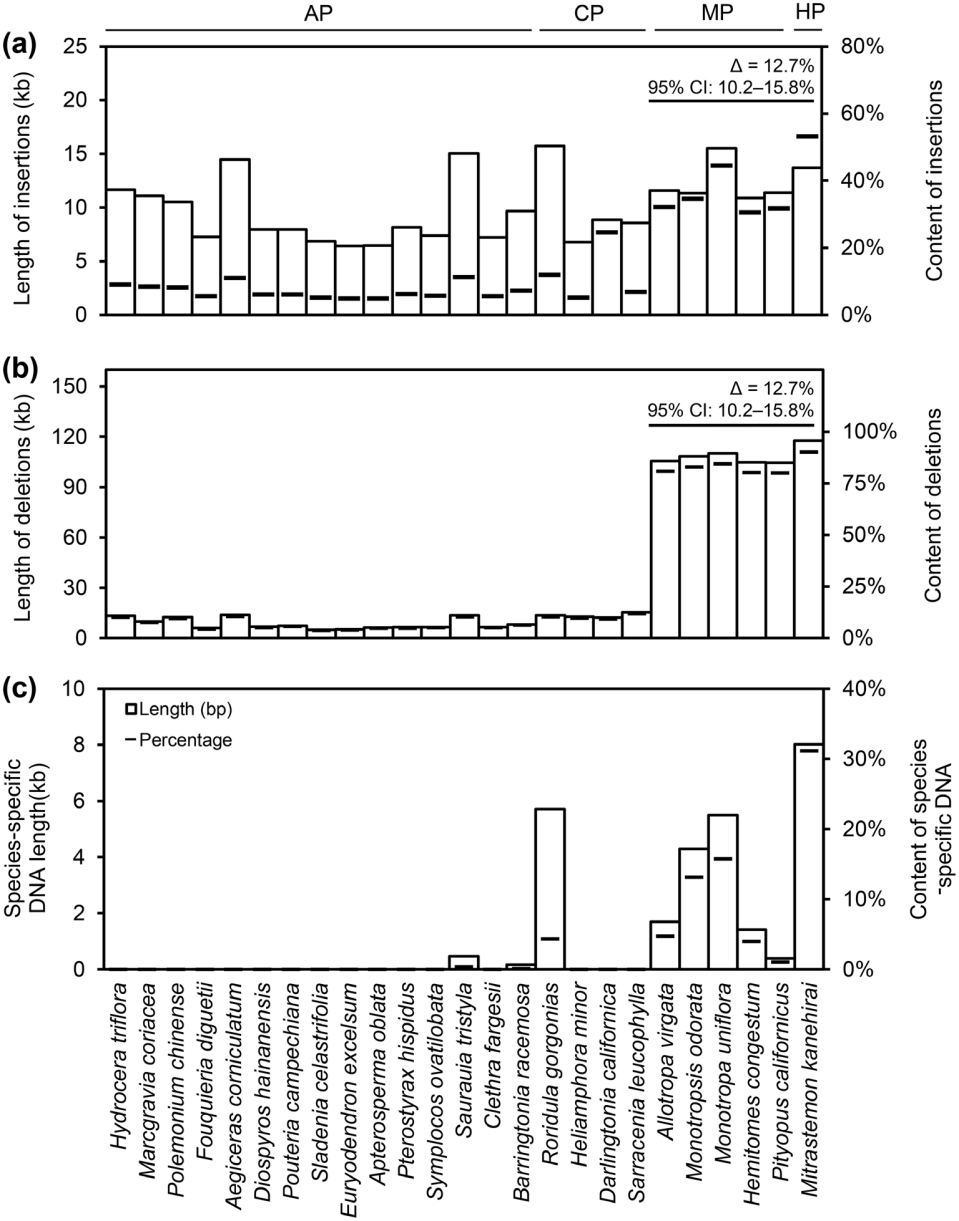
Length and content of insertions (a), deletions (b), and species‐specific DNAs (c) in the Ericales plastomes. Insertion content was calculated as the proportion of insertion length relative to the plastome length of each species, whereas deletion content was calculated as the proportion of deletion length relative to the plastome length of tobacco. CP, MP, and HP denote carnivorous, mycoheterotrophic, and holoparasitic plants, respectively. Comparisons between AP/CP and MP/HP are shown as effect size estimates with 95% confidence intervals derived from the Mann–Whitney test.

To investigate insertion and deletion (indel) dynamics in the RS plastomes, we performed pairwise BLASTN comparisons between Ericales plastomes and the tobacco plastome as a reference. Sequences in Ericales plastomes that lacked homologous regions in the tobacco plastome were classified as Ericales‐specific insertions, whereas sequences in the tobacco plastome without homologs in Ericales were classified as Ericales‐associated deletions. Based on this criterion, our analysis revealed that the insertion length in *Roridula gorgonias* was 1.8 to 2.3 times that in three Sarraceniaceae species, whereas the deletion length difference between *Roridula gorgonias* and the Sarraceniaceae species was less than 1.3‐fold (Figure 6a,b). Furthermore, no significant differences in indel length or proportion were observed between RS plastomes and those of obligate autotrophic plants. However, the indel proportion in carnivorous and obligate autotrophic plants was consistently lower than that in obligate heterotrophic plants (Figure 6a,b). Through intra‐Ericales plastome comparisons, we found that *Roridula gorgonias* exhibits species‐specific DNA fragments not present in Sarraceniaceae species (Figure 6c). These insertions predominantly occur within the *ycf1* gene (Table S2) and are located in the IR region, providing an explanation for the observed IR elongation in *Roridula gorgonias* plastome. Notably, no unique fragments of comparable length to those in *Roridula gorgonias* have been found in autotrophic plants. Even compared with obligate heterotrophic plants, which typically exhibit relatively long insertions, the unique fragment length of *Roridula gorgonias* stands out as remarkable (Figure 6c). This length is exceeded solely by that of the holoparasitic *Mitrastemon* plant and is comparable to the lengths observed in two obligate mycoheterotrophic plants in *Monotropa* and *Monotropsis* genera.

By performing BLASTN searches of species‐specific DNA fragments identified from multiple comparisons against the NCBI *nt* database, we found that 37.8% of the fragments produced high‐confidence hits (*E*‐value < 1e^−5^). Among these, 91.1% of the hits for *Roridula gorgonias* fragments were derived from non‐plant genomes. Notably, fragments showing similarity to non‐plant sequences were also widespread among other heterotrophic nutritional types of plants (Figure S4a; Table S2). At the phylum level, homologous fragments associated with *Arthropoda*, *Chordata*, and *Mollusca* accounted for 59.4% of all BLASTN‐matched classification units (Figure S4b; Table S2). Tracing the intracellular origins of these homologous genomes revealed that most hits corresponding to the unique fragments of *Roridula gorgonias* were located in nuclear genomes, a pattern similar to that observed in other obligate heterotrophic Ericales species (Figure S4c; Table S2).

### 3.4 Gene Expression and RNA Editing

**FIGURE 7:**
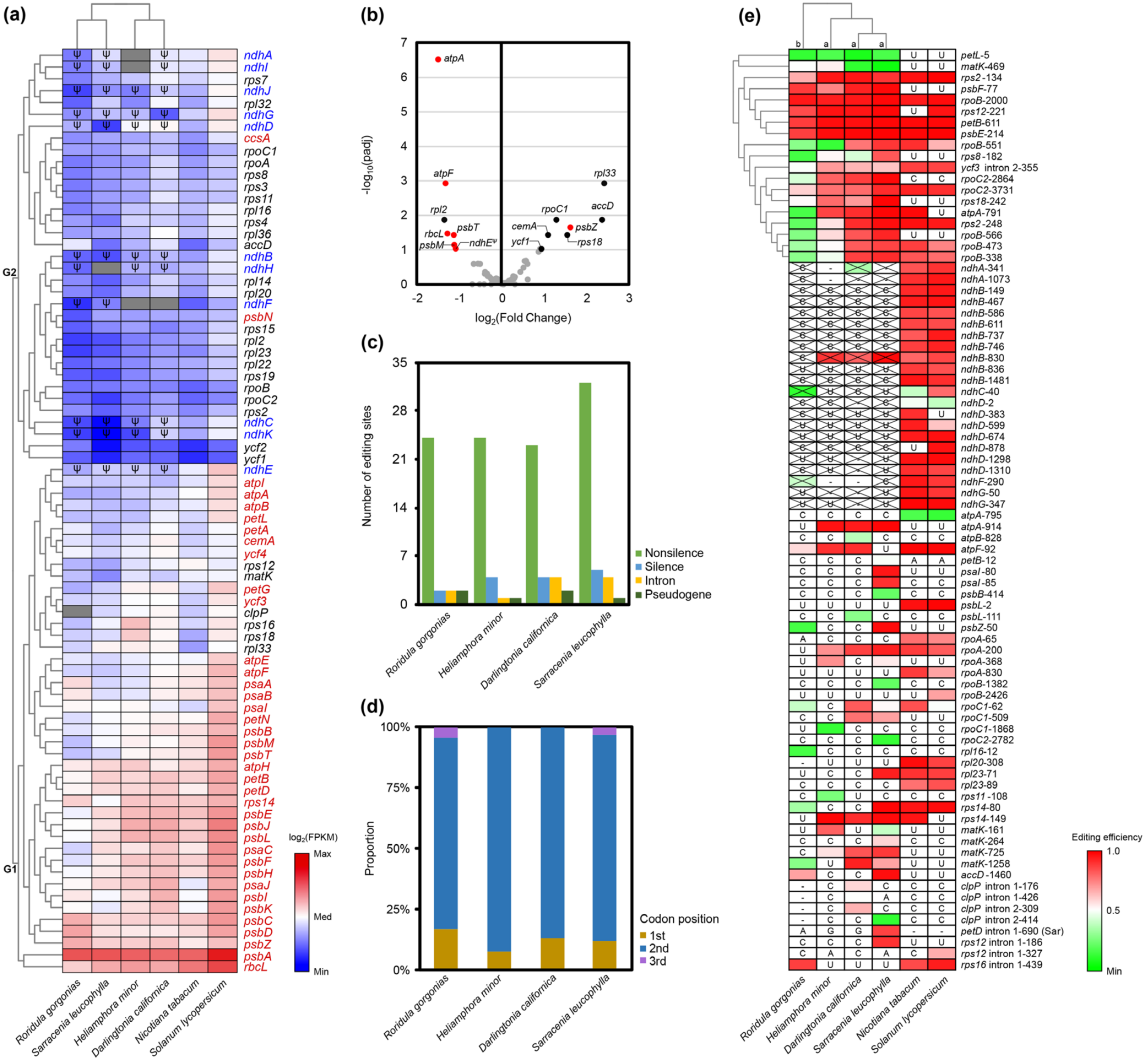
Expression levels and RNA editing efficiency in RS plastid genes. (a) Heatmap of FPKM values in CDS regions. Gray blocks represent ORFs with severe disruption or gene loss, while the symbol ψ denotes pseudogenes. The distance and clustering methods employed are Euclidean distance and Average linkage, respectively. Red gene names indicate photosynthesis‐related genes, blue ones indicate *ndh* genes, and black ones indicate housekeeping genes. (b) Differentially expressed genes (DEGs) in RS carnivorous plants compared to obligate autotrophic plants. Genes with −log_10_(*p*adj) > 1 were considered significant. Red dots represent DEGs related to photosynthesis, while black dots denote housekeeping DEGs. Statistical significance was assessed using DESeq2 with the Wald test, and adjusted *p*‐values were calculated using the Benjamini‐Hochberg procedure (FDR). (c) Distribution of RNA editing events in different genomic sites across RS plastomes. (d) Distribution of RNA editing events in different CDS codon positions across RS plastomes. (e) RNA editing efficiency in plastid genes. Cross marks identify editing sites in pseudogenes, and horizontal lines mark those in deleted genes. Only editing sites common to the four carnivorous plants were clustered. Letters on branches indicate significant differences (one‐way ANOVA followed by LSD post hoc test, *p* value < 0.05). Normality was assessed with the Shapiro–Wilk test, and homogeneity of variance was confirmed by Levene's test (*p* value = 0.53).

Cluster analysis of plastid gene expression levels in RS carnivorous plants revealed that *Heliamphora minor* and *Darlingtonia californica* exhibited similar expression patterns, while *Roridula gorgonias* and *Sarracenia leucophylla* formed a separate cluster (Figure 7a). At the gene level, plastid genes were classified into two expression‐based groups, with group I comprising highly expressed genes and group II comprising genes with consistently lower expression (Figure 7a). Group I mainly comprised photosynthetic genes, whereas Group II primarily included housekeeping genes. Notably, although *ndh* genes have lost their function in RS carnivorous plants, their regions are still transcribed. Except for *ndhE*, all other photosynthetic *ndh* genes clustered into the low‐expression Group I. Compared with autotrophic model plants, genes showing reduced expression in carnivorous plants were predominantly photosynthetic, including *rbcL* and certain *atp* and *psb* genes, whereas genes with increased expression were mostly housekeeping genes (Figure 7b).

The number of RNA editing sites in RS carnivorous plants ranges from 29 to 42 (Table S3), and these editing sites are primarily non‐silent sites within the second codon position of protein‐coding genes (Figure 7c,d). Analysis of 18 shared RNA editing sites across carnivorous plants revealed that *Roridula gorgonias* exhibits significantly lower editing efficiency than three Sarraceniaceae species (ANOVA followed by LSD post hoc test, *p* value < 0.05; Figure 7e). The RNA editing profiles of *Sarracenia leucophylla* and *Darlingtonia californica* , which possess digestion enzymes, cluster together, followed by *Heliamphora minor* and *Roridula gorgonias*, which lack digestion enzymes (Figure 7e). Due to pseudogenization, most of the RNA editing events that commonly occur in the *ndh* genes of obligate autotrophic plants have been lost in RS carnivorous plants (Figure 7e). Among the few remaining editing sites, *ndhB*‐830 stands out as the only site still demonstrating considerable editing activity, while the others show markedly reduced activity compared to their autotrophic counterparts (Figure 7e).

### 3.5 DNA Substitution Rates

**FIGURE 8:**
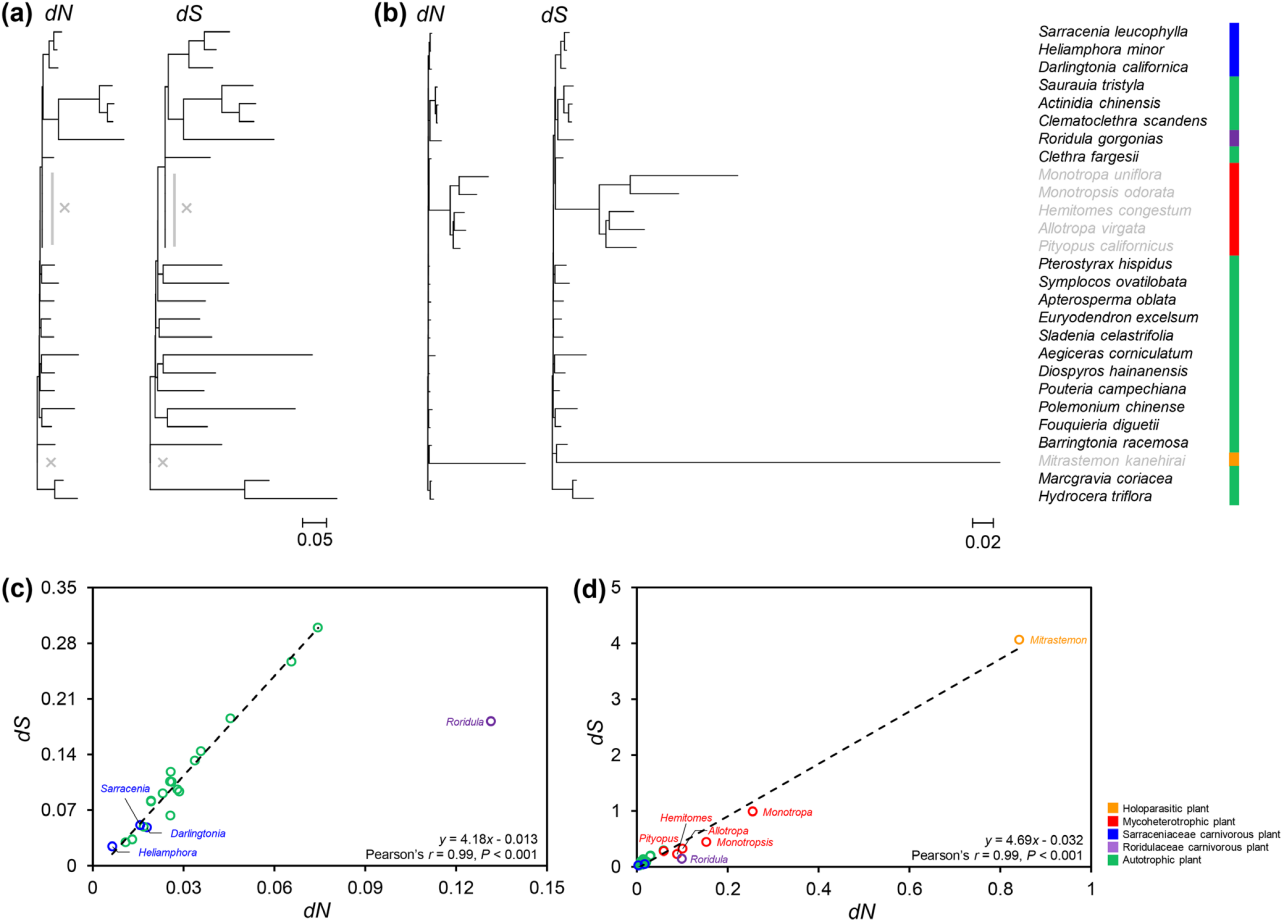
Plastid phylogenomic and correlation between *d*_N_ and *d*_S_ divergence in Ericales. (a) Plastid phylograms of concatenated sequences of 66 shared genes across carnivorous plants and obligate autotrophic plants in Ericales. (b) Plastid phylograms of concatenated sequences of 13 shared genes across carnivorous plants, obligate autotrophic plants and obligate heterotrophic plants in Ericales. (c) Correlation between *d*_N_ and *d*_*S*_ divergence for species branches in phylograms shown in (a). Linear regression analyses in c included all data points in (a) except for *Roridula gorgonias* terminal branch value. (d) Correlation between *d*_N_ and *d*_*S*_ divergence for species branches in phylograms shown in (b).

To investigate the evolutionary patterns of plastid genes in RS carnivorous plants, a total of 66 common genes shared by carnivorous plants and obligate autotrophic plants were examined. An analysis of substitution rates of these shared gene sequences revealed that *Roridula gorgonias* displays a decoupling of its substitution rates (Figure 8a,b). When *Roridula gorgonias* was excluded from the analysis, a striking correlation emerged between *d*_N_ rates and *d*_S_ rates across all branches of the phylogenetic tree. The average slope of the linear regression between *d*_S_ and *d*_N_ was found to be 4.2 (Figure 8c), which stands in sharp contrast to the ratio of 1.4 for *Roridula gorgonias*. Furthermore, when obligate heterotrophic plants are included in the comparison, only 13 protein‐coding genes are found to be shared among the Ericales. In contrast to these obligate heterotrophic plants, *Roridula gorgonias* continues to diverge from the expected trendline of *d*_N_ and *d*_S_ (Figure 8d).

**FIGURE 9:**
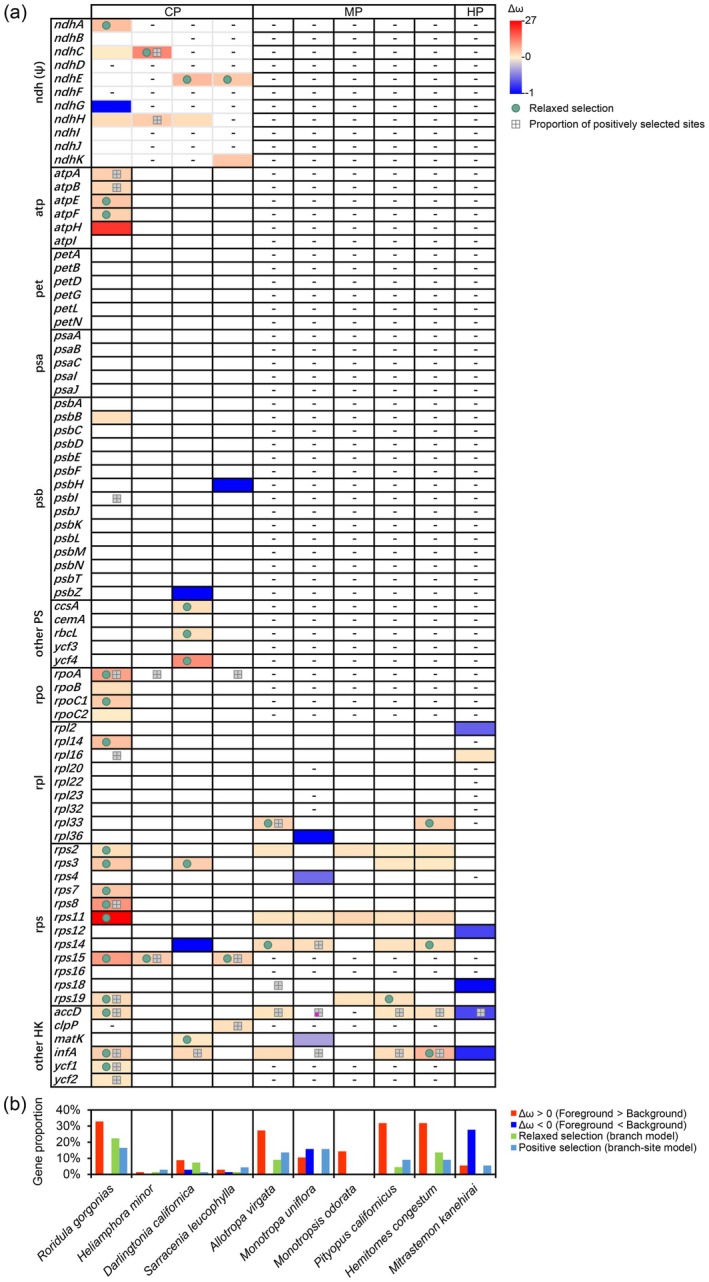
Selection patterns of plastid genes in heterotrophic lineages of Ericales. (a) Summary of branch‐specific selection signals and statistics of plastid genes in heterotrophic plants of Ericales. Obligate autotrophic Ericales species (Table S1) were designated as background branches. Codon‐based tests were performed by comparing a null one‐ratio model across the tree (M0) with an alternative two‐ratio model allowing the foreground branch to evolve independently (b_free). Only genes showing significant differences between b_free and M0 (LRT, *p* < 0.05) are color‐coded. The heatmap depicts the relative change in selective pressure between foreground and background branches, quantified as Δω = (ω_fg − ω_bg)/ω_bg, using a red–yellow–blue gradient, where red indicates Δω > 0 (foreground ω > background ω) and blue indicates Δω < 0 (foreground ω < background ω). Green‐filled circles denote genes inferred to be under relaxed selection, defined as significant b_free vs. M0 results (*p* < 0.05) but no significant deviation of the foreground branch from neutrality in comparisons with b_neut (*p* ≥ 0.05). Squares indicate the proportion of positively selected sites inferred from branch‐site models (bsA vs. bsA1), with fully gray squares representing BEB site ratios < 0.25% and squares containing one pink quadrant indicating BEB site ratios between 0.25% and 0.50%. CP, MP, and HP denote carnivorous, mycoheterotrophic, and holoparasitic plants, respectively. Genes with non‐significant results, extreme ω estimates (ω ≥ 10 or ω ≤ 0.001), or otherwise unreliable estimates are shown without color. “–” indicates pseudogenized or missing genes. (b) Proportion of plastid genes showing different selective patterns in heterotrophic Ericales. *ndh* genes were excluded from these statistics.

Selective analyses revealed that *Roridula gorgonias* exhibits widespread signatures of altered selective pressures across multiple functional categories of plastid genes, including RNA polymerase genes, ATP synthase genes (excluding *atpI*), and a substantial proportion of small ribosomal subunit protein–coding genes as well as other housekeeping genes (Figure 9a). Relative to obligate autotrophic background branches, all of these genes showed evidence of relaxed constraint. Further comparisons between the b_free and b_neut models indicated that 68.2% of these genes were inferred to be under relaxed selection, and 45.5% contained positively selected sites based on branch‐site analyses (Figure 9a). Within the RS clade, *Roridula gorgonias* displayed the highest proportions of genes showing relaxed constraint, relaxed selection, and evidence of positive selection, exceeding not only Sarraceniaceae lineages but also obligate heterotrophic plants (Figure 9b).

Among Sarraceniaceae species, the selective pressures acting on core photosynthetic genes (*atp*, *pet*, *psa*, and *psb*) and the housekeeping gene *rpl* did not differ significantly from those observed in obligate autotrophic plants. Notably, *psbH* and *psbZ* were inferred to be under strengthened constraint. *Heliamphora minor* showed the highest overall similarity to obligate autotrophic species, with only *rps15* exhibiting evidence of relaxed selection. In contrast, *Darlingtonia californica* exhibited the highest proportions of genes under relaxed constraint, strengthened constraint, and relaxed selection, while showing the lowest proportion of genes containing positively selected sites (Figure 9b). In the holoparasitic plant *Mitrastemon kanehirai*, five out of six housekeeping genes with significant constraint status showed evidence of strengthened constraint. In the mycoheterotrophic plant *Monotropa uniflora* , three out of five housekeeping genes with significant constraint status were inferred to be under strengthened constraint.

For *ndh* pseudogenes, the ORFs of *ndhA*, *ndhC*, and *ndhH* in *Roridula gorgonias*, as well as all *ndh* genes in Sarraceniaceae species, exhibited signatures of relaxed constraint. In contrast, the ORF of *ndhG* in *Roridula gorgonias* showed evidence of strengthened constraint, whereas the ORFs of the remaining *ndh* genes did not show significant deviations from background selective pressures (Figure 9a).

Substitution rate analysis found the majority of genes in *Roridula gorgonias* show higher *d*_N_ (75.8%–84.8%) and exhibit elevated *d*_S_ values (56.1%–71.2%) compared to those in the three Sarraceniaceae species (data not shown). Compared to obligate autotrophic plants, in RS carnivorous plants, the *d*_N_ rates of *atpI*, *psbF*, *psbK*, *ycf4* and *rps7*, and the *d*_S_ rates of *psbE*, *psbZ*, *rps7*, *rps11*, *rps14* and *rps18*, along with substitution rates of *trnA*‐UGC, *trnG*‐UCC, *trnV*‐UAC, *trnW*‐CCA, are significantly higher (Figure S5). Conversely, only the *d*_N_ rate of *ycf3*, *petD* and the *d*_S_ rates of *ccsA*, *rpl23* and *rps12* are significantly lower. Moreover, the interquartile range of *d*_N_ for *atpF*, *petN*, *psbM*, *psbT*, *rps11*, *accD*, *ycf1*, and *ycf2* in carnivorous plants is over twice that in obligate autotrophic plants (Figure S5). Compared with obligate heterotrophic plants, all shared plastid genes in Ericales from carnivorous and obligate autotrophic plants exhibit significantly lower substitution rates (Mann–Whitney test, all *p* values < 0.001; data not shown).

## 4 Discussion

### 4.1 Annotation of Plastid Genes in Heterotrophic Plants

Plastid gene annotation involves the systematic identification of protein‐coding, rRNA, and tRNA genes. In principle, experimental molecular biology approaches can be used to directly confirm gene presence and determine their precise positions within the plastome. However, such experimental validation is extremely cumbersome, time‐consuming, and labor‐intensive, and in practice, only a few species—such as *Nicotiana tabacum* and *Arabidopsis thaliana* —are amenable to plastome transformation and comprehensive functional studies that allow direct verification of gene function (Narra et al. 2025). For the majority of species, direct experimental validation of plastid gene annotations remains infeasible. Currently, plastid gene annotations are primarily based on homology‐based DNA sequence alignment, with protein‐coding genes further verified through ORF analysis and tRNA genes validated by secondary structure prediction. Plastid reference genes from closely related species are typically inferred through alignment with model plant genes; however, repeated reliance on such inferred sequences has led to variable annotation quality in GenBank, complicating the identification of perfectly homologous reference genes. Annotation is further challenged in heterotrophic plants, whose plastid genes often exhibit substantial sequence divergence relative to their autotrophic relatives.

To overcome these challenges, tobacco plastid genes, which are well‐characterized and extensively validated, were used as a reference for homology‐based alignment in annotating plastid genes in carnivorous Ericales. Based on coverage patterns commonly observed in autotrophic Ericales, we set a 70% threshold to reduce subjective bias and guide gene annotation (Figure 1). Although this approach does not achieve complete precision, it allows a systematic assessment of plastid gene presence and integrity in the RS clade. During annotation, we further adopted the principle that if any single *ndh* gene shows pseudogenization or is lost, all *ndh* genes are annotated as pseudogenes, regardless of ORF integrity. This principle is supported by experimental evidence indicating that once any *ndh* gene becomes a pseudogene, the NDH complex is no longer assembled (Burrows et al. 1998; Horváth et al. 2000; Yamamoto et al. 2021; Strand et al. 2024), with no evidence of functional transfer to the nuclear genome, and these subunits have no known roles in other physiological processes.

We also explored additional molecular evidence to evaluate potential gene pseudogenization, such as whether pseudogenization affects transcriptional activity or leads to pronounced relaxation of selective constraints. Based on our results, regions of *ndh* genes annotated as pseudogenes are still transcribed (Figure 7), either due to passive co‐transcription within plastid operons or reflecting residual or redundant transcriptional activity, which alone does not indicate pseudogenization status. While most *ndh* ORFs exhibit relaxed selective constraints, the level of selective pressure acting on them is comparable to that of many housekeeping genes under similar constraints, with the *ndhG* sequence in *Roridula gorgonias* even showing relatively stronger constraint. This lack of pronounced relaxation of selective constraints may also reflect the retention of these *ndh* sequences in the plastome for structural or regulatory redundancy, despite their complete loss of physiological function. These observations indicate that transcriptional activity and selection pressure alone cannot reliably diagnose plastid gene pseudogenization.

RNA editing serves as a post‐transcriptional mechanism for correcting deleterious mutations (Lukeš et al. 2021). In RS carnivorous plants, many cytosine sites in *ndh* genes fail to undergo RNA editing when compared to obligate autotrophic model plants (Figure 7e). This lack of RNA editing may reflect pseudogenization; however, richer plastid transcriptomic data from the RS clade's sister autotrophic lineages are needed for further comparative validation. In addition, the distribution of RNA editing sites across different plastid genes is highly uneven (Table S3) (Zhang et al. 2023), making its practical utility quite limited. Ultimately, direct experimental approaches, such as plastome transformation, remain the gold standard for the definitive validation of gene function and pseudogenization. Our approach, comparing against tobacco and incorporating coverage information, provides an improvement over standard bioinformatic‐based alignment.

### 4.2 Plastome Variation and Evolutionary Dynamics Across RS Taxa

Compared with obligate autotrophic plants, RS carnivorous plants consistently display several characteristic features including an expansion of the IR region (Table S1), pseudogenization or loss of *ndh* genes (Figure 2), structural rearrangements within the *ndh* gene cluster (Figure 3), and reduced expression levels of photosynthetic genes (Figure 7b). IR expansion has also been reported in *Drosera erythrorhiza* and *Drosera rotundifolia* , whereas a reduction in IR length has been observed in *Dionaea muscipula* (Nevill et al. 2019). In contrast, carnivorous plants from the Lentibulariaceae show IR lengths comparable to those of obligate autotrophic species, without apparent expansion or contraction (Wicke et al. 2014). These observations indicate that IR size exhibits no consistent evolutionary trend across carnivorous lineages.

Plastid *ndh* genes encode subunits of the NADH dehydrogenase complex, which has been proposed to optimize photosynthesis under fluctuating environmental conditions and various abiotic stresses (Martín and Sabater 2010; Peredo et al. 2013). Losses and structural rearrangements of *ndh* genes have been documented in all reported carnivorous plants (Wicke et al. 2014; Nevill et al. 2019; Fu et al. 2023), indicating that *ndh* degeneration is a common feature in these lineages. Experimental plastid transformation studies have demonstrated that *ndh* genes can be completely disrupted to homoplasmy in tobacco without causing lethality (Burrows et al. 1998; Horváth et al. 2000; Yamamoto et al. 2021; Strand et al. 2024), supporting the view that the NDH complex functions as an auxiliary, rather than indispensable, component of photosynthesis. In our study, *ndh* pseudogenes mainly clustered with lowly expressed housekeeping genes rather than with highly expressed photosynthetic genes (Figure 7a), consistent with their pseudogenized status. Furthermore, because *ndh* genes tend to undergo coordinated pseudogenization as an integrated functional unit (Burrows et al. 1998; Horváth et al. 2000; Yamamoto et al. 2021; Strand et al. 2024), their degeneration does not appear to correspond in a measurable way to the intensity of carnivory.

In terms of selection intensity, a notable decline in conservative selection has been observed in genes associated with photosynthesis, metabolism, and plastid‐encoded polymerases in carnivorous plants from the Lentibulariaceae, Droseraceae, and Caryophyllales families, when compared to obligate autotrophic plants (Wicke et al. 2014; Nevill et al. 2019). This phenomenon is believed to be closely linked to reduced net photosynthesis rates, a reliance on alternative nutrient acquisition strategies such as organic carbon uptake, and compromised DNA repair mechanisms. However, excluding the exceptional case of *Roridula gorgonias*, core photosynthetic genes and *rpo* genes in the three examined Sarraceniaceae species do not exhibit clear signatures of relaxed selection, and among other photosynthesis‐related genes, relaxed selection was detected only in *Darlingtonia californica* (Figure 9a). These findings indicate that the impact of carnivory on selective constraints acting on plastid genes is not universal. Moreover, no consistent correlation is observed between plastid gene selection intensity and the degree of carnivory across RS taxa (Figure 9b).

Studies on plastid gene expression in carnivorous plants at the transcriptional level are limited. In our analysis, the reduced expression levels of photosynthetic genes in carnivorous plants are consistent with their decreased photosynthetic capacity (Figure 7b). From the perspective of a more refined gradient of carnivory, the transcriptional patterns of RS carnivorous plants do not consistently correspond to their degree of carnivorous capability. In contrast, clustering relationships based on RNA editing efficiency exhibit some correlation with carnivory (Figure 7e), although it remains challenging to determine whether this relationship is directly caused by carnivory itself.

### 4.3 Ecological Context and Plastome Evolution in the RS Clade

Carnivorous plants generally exhibit plastome degradation, yet this degradation does not align clearly with carnivorous specialization, even within Sarraceniaceae with a divergence of ~44 Mya (Kumar et al. 2017). Plastome degradation is strongly linked to a decrease in photosynthetic capacity (Braukmann et al. 2017; Wicke and Naumann 2018). Studies have shown that both aquatic and terrestrial carnivorous species display lower photosynthetic nutrient‐use efficiency and reduced photosynthesis per leaf area compared with non‐carnivorous plants, with estimated reductions of approximately 1%–5% (Méndez and Karlsson 1999; Ellison 2006; Adamec 2010).

On one hand, carbon acquired through carnivory can play a role in the reduction of photosynthetic capacity in carnivorous plants. Experimental evidence shows that carnivorous plants acquire a small fraction of their carbon from prey. For example, δ^13^C enrichment experiments in *Drosera capensis* and *Drosera regia* indicate that prey‐derived carbon contributes at most ~0.29% of total plant carbon (Lin et al. 2025). In *Dionaea muscipula* , labeled amino acid carbon experiments further demonstrate that prey‐derived carbon is mainly used for local respiration within traps (Fasbender et al. 2017), indicating that prey carbon does not replace photosynthesis as the main carbon source. On the other hand, under natural conditions, long‐term nutrient limitation likely plays an important role in photosynthetic capacity reducing. Nutrient scarcity, particularly low nitrogen availability, limits the synthesis of key photosynthetic proteins such as Rubisco and components of the photosynthetic electron transport chain, leading to lower photosynthetic rates (Adamec 2010; Capó‐Bauçà et al. 2020). This scarcity also promotes conservative growth strategies, including low leaf nitrogen content, slow growth, and preferential allocation of resources to carnivorous organs rather than photosynthetic tissues (Ellison 2006; Pavlovič and Saganová 2015).

*Roridula gorgonias* represents a more extreme case. Despite its relatively weak carnivorous capacity, it exhibits pronounced, lineage‐specific plastome remodeling. Relaxation of selective constraints affects extensive regions of the plastome, including *atp* and housekeeping genes; even relative to obligate heterotrophic plants, these genes show a striking degree of relaxation (Figure 9). Meanwhile, approximately 45.5% of these relaxed genes harbor positively selected sites, representing a rare instance of concurrent relaxation and adaptive evolution that distinguishes this species from its Sarraceniaceae relatives. Additionally, its plastome exhibits substantial DNA insertions and reduced RNA editing efficiency at conserved sites (Figures 6 and 7e). These distinctive modifications in *Roridula gorgonias* likely reflect the impact of particularly severe ecological pressures. The species inhabits nutrient‐poor, acidic mountain regions in South Africa, where recurrent, high‐intensity wildfires periodically affect its habitat (Ellison and Adamec 2018). Under these conditions, the plastome may accumulate mutations that are tolerated, and some of these changes could be adaptive. By contrast, Sarraceniaceae species primarily occur in temperate to subtropical regions of North and South America, where they do not face comparably severe environmental stresses (Ellison and Adamec 2018).

From an ecological perspective, plastome evolution in the RS clade underscores that plastome dynamics cannot be attributed solely to carnivory, illustrating how long‐term nutrient adaptation and lineage‐specific ecological pressures can potentially amplify plastome remodeling. To date, continuous gradients of carnivory have only been documented within the RS clade, preventing direct testing of these patterns in independent lineages. The RS clade comprises approximately 34 species, exhibiting substantial variation in population size, geographic distribution, and habitat characteristics (Ellison and Adamec 2018; Simpson 2019). Future studies should integrate population‐level plastome sampling, nuclear genome data, and ecological habitat information, coupled with experimental validation of the causal relationships among reduced photosynthetic efficiency, relaxed selective constraints, and decreased RNA editing efficiency under environmental and nutrient stresses. Such approaches are essential for understanding the intricate evolutionary history of the genomes of carnivorous plants.

### 4.4 Can Close Contact With Animals Introduce Foreign Nucleotides Into the RS Plastomes?

Plant genomes frequently integrate foreign DNA via horizontal gene transfer (HGT), with donors spanning bacterial/archaeal domains (Soucy et al. 2015). In mosses, this process even involves metazoan‐derived actinoporin‐like genes that confer desiccation tolerance (Hoang et al. 2009). Conversely, the whitefly ( *Bemisia tabaci* ) acquired the plant‐derived BtPMaT1 gene, enabling detoxification of host‐produced phenolic glycosides (Xia et al. 2021). Carnivorous plants, particularly those secreting digestive enzymes, exhibit heightened exposure to animal‐derived nucleotides through direct trophic interactions. This raises the hypothesis: could animal‐to‐plant HGT events contribute to the evolutionary innovation of carnivorous traits?

At least, our exploration of the potential sources of unique fragments in RS plastomes does not support this view. Given the current limitations in genomic data across the tree of life, definitive attribution of carnivorous plant plastome‐unique regions to animal donors remains challenging. However, quantitative assessment of these fragments reveals that: (1) digestive carnivorous plants exhibit comparable levels of insertion and species‐specific DNA content to obligate autotrophs (Figure 6a,c; Table S2), and (2) non‐digestive *Roridula gorgonias* and obligate heterotrophs contain potentially animal‐derived sequences, whereas no such sequences were detected in digestive carnivorous plants (Figure S4). These results indicate that mere physical proximity to animals does not necessarily elevate nucleotide incorporation rates, and digestive enzyme secretion is not a primary factor influencing foreign DNA acquisition in plant plastomes.

Furthermore, genomic analyses to date provide no evidence for the horizontal transfer of animal‐derived genes into the nuclear genomes of carnivorous plants to facilitate the evolution of rapid prey‐capture mechanisms (Hedrich and Fukushima 2021). Additionally, animal DNA has not been detected within the mitochondrial genomes of carnivorous plants, despite these genomes being recognized for their propensity towards frequent HGT (Matos et al. 2022; Silva et al. 2023). These findings contradict the gene‐hijacking hypothesis and instead support the adaptive evolution model proposed by Hedrich and Fukushima (2021), wherein nutrient limitation in ancestral ecosystems drove the exaptation of defensive traits into active carnivory through incremental phenotypic innovations.

According to the adaptive evolution model, this evolutionary trajectory appears to be primarily driven by changes in the nuclear genome, particularly involving the recruitment of pre‐existing genes and the regulatory rewiring of pathways related to defense, stress responses, and protein secretion (Renner and Specht 2011; Fukushima et al. 2017). In contrast, no direct evidence currently supports the involvement of endogenous plastid or mitochondrial genes in the origin or functional development of plant carnivory.

### 4.5 The Plastome of Carnivorous Plants Exemplifies a Predominantly Degraded Type

Through investigations into plastome degradation in mycoheterotrophic plants and synthesis of existing theoretical frameworks, Graham et al. (2017) proposed that initial loss of the NDH complex frequently triggers irreversible cascades of photosynthetic gene loss. Genes with secondary functions may exhibit asynchronous retention patterns, with delayed elimination of non‐bioenergetic genes accounting for the long‐term persistence of plastomes in heterotrophic organisms. By studying plastome degradation in parasitic plants, Wicke et al. (2016) proposed a similar model for parasitic plants, emphasizing gene loss order, nonfunctionalization, physical reductions, lifestyle‐specific evolutionary rate shifts, and declining plastid GC content.

Given the consistent observation that plastid gene loss in our studied RS species and all other documented carnivorous plants (Wicke et al. 2014; Nevill et al. 2019; Fu et al. 2023) is confined primarily to *ndh* genes, if carnivorous plants are placed within the plastome degradation models of mycoheterotrophic and parasitic plants, carnivorous species are merely at an early evolutionary stage of plastome degradation. This conclusion also remains valid beyond the predictive scope of these models. As our study has clearly demonstrated, when compared to obligate heterotrophic plants, RS carnivorous plants obviously exhibit higher plastomic conservation, as demonstrated by comparisons of genome length (Table S1), GC content (Figure 4), short repeat content (Figure 5), and other structural characteristics. In studies on other carnivorous plants, these plants also show a higher degree of similarity to obligate autotrophic plants compared to obligate heterotrophic plants (Shyu 2013; Wicke et al. 2014; Braukmann et al. 2017; Nevill et al. 2019). As discussed above, carbon obtained through carnivory only partially supplements the plant's carbon requirements, so carnivorous plants remain highly dependent on photosynthetic carbon fixation, which explains the restricted plastome degradation observed in carnivorous species. This contrasts with parasitic or mycoheterotrophic species, which can acquire carbon directly and extensively, sometimes even fully substituting for photosynthesis (Pavlovič and Saganová 2015; Tĕšitel et al. 2018).

## Author Contributions

**Shengxin Chang:** conceptualization (equal), data curation (equal), formal analysis (equal), funding acquisition (equal), project administration (equal), visualization (equal), writing – original draft (equal). **Peng Wang:** software (equal), validation (equal), writing – review and editing (equal). **Wei Han:** visualization (equal), writing – review and editing (equal). **Baiyin Yu:** writing – review and editing (equal). **Chunxia Li:** conceptualization (equal), validation (equal), visualization (equal), writing – review and editing (equal).

## Funding

This work was financially supported by the National Natural Science Foundation of China (31701947), the Science and Technology Plan Project of Shaoguan Science and Technology Bureau (220607104530687), Talent Scientific Research Funding of Shaoguan University, and the China Scholarship Council Scholarship (202003260016 and 202108440316).

## Ethics Statement

The authors confirm that all methods followed relevant guidelines and regulations. No endangered wild plant species were used, and no special permits were needed for sample collection. We complied with relevant institutional, national, and international guidelines and legislation for plant study.

## Consent

The authors have nothing to report.

## Conflicts of Interest

The authors declare no conflicts of interest.

## Supporting information


**Figure S1:** Multiple sequence alignment of the *ndhB* (a) and *ndhC–ndhK–ndhJ* (b) genes between the Roridulaceae–Sarraceniaceae (RS) carnivorous plant and its closely related autotrophic plant *Clethra fargesii*. Pure blue arrows indicate open reading frames (ORFs), while lighter‐shaded regions represent non‐ORF areas that are homologous to the gene sequences of the obligate autotrophic plant. The symbol * indicates stop codons. The red, blue, yellow, and green lines above the sequence bars for each species correspond to the ACGT bases at polymorphic sites, respectively. The numerical segments enclosed in parentheses at the beginning and end of the sequences represent their actual positions on the plastome of *C. fargesii* .
**Figure S2:** Comparison of the junctions between the large single copy (LSC), small single copy (SSC), and inverted repeat (IR) regions in Ericales plastomes. JLB: Junction between the LSC and IRb. JSB: Junction between the IRb and SSC. JSA: Junction between the SSC and IRa. JLA: Junction between the IRa and LSC. The symbol ψ denotes pseudogenes.
**Figure S3:** Impact of varying Blastn *E*‐values on short repeat analysis in Ericales plastomes. (a) Short repeat content of plastomes at different Blastn *E*‐values. (b) Slope values derived from the regression of short repeat content against log_10_(*E*‐value), with error bars representing 95% confidence intervals for each plastome. Horizontal lines indicate differences between AP/CP and MP/HP. AP, CP, MP, and HP denote obligate autotrophic, carnivorous, mycoheterotrophic, and holoparasitic plants, respectively.
**Figure S4:** (a) Proportions of best hits from different kingdoms in the NCBI core nucleotide database for species‐specific DNA fragments in Ericales plastomes. Different kingdoms are indicated by distinct colors, and unmatched fragments are shown as empty boxes. (b) Proportions of best hits from different phyla based on the results in panel (a). (c) Proportions of best hits from different genomic compartments based on the results in panel (a). AP, CP, MP, and HP denote obligate autotrophic, carnivorous, mycoheterotrophic, and holoparasitic plants, respectively.
**Figure S5:** Substitution rates of shared plastid genes across carnivorous and obligate autotrophic plants in Ericales, estimated relative to their tobacco homologs. (a) *d*_N_ values for shared protein‐coding genes. (b) *d*_S_ values for shared protein‐coding genes. (c) Substitution rates of shared rRNA genes, tRNA genes, and introns. The height of the box represents the range from the lower to the upper quartile, while the horizontal line inside the box denotes the median. Points on the scatter plot represent actual data points from each genome, including outliers and extreme values. Asterisks indicate statistically significant differences analyzed using Wilcoxon rank sum tests, with *, **, and *** representing *p*‐values < 0.05, < 0.01, and < 0.001, respectively.
**Table S1:** The Ericales plastomes involved in the present study.
**Table S2:** BLASTN hits of species‐specific DNA regions of Ericales plastomes against the NCBI core nucleotide database (nt; database update: 24 December 2025), using BLASTN with the following parameters: word size = 11; match/mismatch = 2/−3; gap open = 5, gap extension = 2; *E*‐value < 1e−5.
**Table S3:** Plastid RNA editing sites of RS carnivorous plants and two autotrophic model plants.

## Data Availability

Complete plastome sequences of four RS species have been submitted to the GenBank database (https://www.ncbi.nlm.nih.gov/genbank/) with the accession numbers “PP273159.1–PP273162.1”. Clean RNA‐seq data have been submitted to the NCBI SRA database (http://www.ncbi.nlm.nih.gov/sra/) with the accession numbers “SRR27829005‐SRR27829008”. The automated bioinformatic pipeline used in this study is publicly available at GitHub—Plastome Analysis Pipeline with DOI: https://doi.org/10.5281/zenodo.18593566. Raw and processed data have been uploaded to Zenodo with DOI: https://doi.org/10.5281/zenodo.18323667.
