# Supplementary material for: Plastid Genome Evolution Across the Roridulaceae–Sarraceniaceae Clade (Ericales) in Relation to Carnivorous Strategies
Source: Ecol Evol. 2026 Mar 26;16(4):e73333. doi: 10.1002/ece3.73333 (PMC13107276; doi:10.1002/ece3.73333)
Supplement: Supplementary file 1 — Figure S1: Multiple sequence alignment of the ndhB (a) and ndhC–ndhK–ndhJ (b) genes between the Roridulaceae–Sarraceniaceae (RS) carnivorous plant and its closely related autotrophic plant Clethra fargesii. Pure blue arrows indicate open reading frames (ORFs), while lighter‐shaded regions represent non‐ORF areas that are homologous to the gene sequences of the obligate autotrophic plant. The symbol * indicates stop codons. The red, blue, yellow, and green lines above the sequence bars for each species correspond to the ACGT bases at polymorphic sites, respectively. The numerical segments enclosed in parentheses at the beginning and end of the sequences represent their actual positions on the plastome of C. fargesii . Figure S2: Comparison of the junctions between the large single copy (LSC), small single copy (SSC), and inverted repeat (IR) regions in Ericales plastomes. JLB: Junction between the LSC and IRb. JSB: Junction between the IRb and SSC. JSA: Junction between the SSC and IRa. JLA: Junction between the IRa and LSC. The symbol ψ denotes pseudogenes. Figure S3: Impact of varying Blastn E‐values on short repeat analysis in Ericales plastomes. (a) Short repeat content of plastomes at different Blastn E‐values. (b) Slope values derived from the regression of short repeat content against log10(E‐value), with error bars representing 95% confidence intervals for each plastome. Horizontal lines indicate differences between AP/CP and MP/HP. AP, CP, MP, and HP denote obligate autotrophic, carnivorous, mycoheterotrophic, and holoparasitic plants, respectively. Figure S4: (a) Proportions of best hits from different kingdoms in the NCBI core nucleotide database for species‐specific DNA fragments in Ericales plastomes. Different kingdoms are indicated by distinct colors, and unmatched fragments are shown as empty boxes. (b) Proportions of best hits from different phyla based on the results in panel (a). (c) Proportions of best hits from different genomic compartments base [file ECE3-16-e73333-s001.docx]

Supplementary Material

**Plastid genome evolution across the Roridulaceae–Sarraceniaceae clade (Ericales) in relation to carnivorous strategies**

Shengxin Chang^1,2^, Peng Wang^3^, Wei Han^1,2^, Baiyin Yu^1,2^, Chunxia Li^1,2*^

**^1^**School of Biology and Agriculture, Shaoguan University, Daxue Road NO. 288, Shaoguan, Guangdong 512005, China.

**^2^** Guangdong Provincial Key Laboratory of Northern Guangdong Food and Drug Resources Utilization and Protection, Daxue Road NO. 288, Shaoguan, Guangdong 512005, China.

**^3^**Institute of Tropical Crop Genetic Resources, Chinese Academy of Tropical Agricultural Sciences, No. 4 Xueyuan Road, Haikou, Hainan 571101, China.

***Correspondence:**

Chunxia Li

LeeOlivia2015@outlook.com

**Fig. S1** Multiple sequence alignment of the *ndhB* (**a**) and *ndhC-ndhK-ndhJ* (**b**) genes between the Roridulaceae–Sarraceniaceae (RS) carnivorous plant and its closely related autotrophic plant *Clethra fargesii*. Pure blue arrows indicate open reading frames (ORFs), while lighter-shaded regions represent non-ORF areas that are homologous to the gene sequences of the obligate autotrophic plant. The symbol * indicates stop codons. The red, blue, yellow, and green lines above the sequence bars for each species correspond to the ACGT bases at polymorphic sites, respectively. The numerical segments enclosed in parentheses at the beginning and end of the sequences represent their actual positions on the plastome of *C. fargesii*.

**Fig. S2** Comparison of the junctions between the large single copy (LSC), small single copy (SSC), and inverted repeat (IR) regions in Ericales plastomes. JLB: Junction between the LSC and IRb. JSB: Junction between the IRb and SSC. JSA: Junction between the SSC and IRa. JLA: Junction between the IRa and LSC. The symbol ψ denotes pseudogenes.

**Fig. S3** Impact of varying Blastn *E*-values on short repeat analysis in Ericales plastomes. **a** Short repeat content of plastomes at different Blastn *E*-values. **b** Slope values derived from the regression of short repeat content against log_10_(*E*-value), with error bars representing 95% confidence intervals for each plastome. Horizontal lines indicate differences between AP/CP and MP/HP. AP, CP, MP, and HP denote obligate autotrophic, carnivorous, mycoheterotrophic, and holoparasitic plants, respectively.

**Fig. S4** **a** Proportions of best hits from different kingdoms in the NCBI core nucleotide database for species-specific DNA fragments in Ericales plastomes. Different kingdoms are indicated by distinct colors, and unmatched fragments are shown as empty boxes. **b** Proportions of best hits from different phyla based on the results in panel **a**. **c** Proportions of best hits from different genomic compartments based on the results in panel **a**. AP, CP, MP, and HP denote obligate autotrophic, carnivorous, mycoheterotrophic, and holoparasitic plants, respectively.

**Fig. S5** Substitution rates of shared plastid genes across carnivorous and obligate autotrophic plants in Ericales, estimated relative to their tobacco homologs. **a** *d*_N_ values for shared protein-coding genes. **b** *d*_S_ values for shared protein-coding genes. **c** Substitution rates of shared rRNA genes, tRNA genes, and introns. The height of the box represents the range from the lower to the upper quartile, while the horizontal line inside the box denotes the median. Points on the scatter plot represent actual data points from each genome, including outliers and extreme values. Asterisks indicate statistically significant differences analyzed using Wilcoxon rank sum tests, with *, **, and *** representing *P*-values <0.05, <0.01, and <0.001, respectively.

**Table S1.** The Ericales plastomes involved in the present study

| **Species name** | **Family** | **Nutritional**  **type** | **Genbank**  **NO.** | **Plastome size (bp)** | **IR length**  **(bp)** | **NR length (bp)** |
| --- | --- | --- | --- | --- | --- | --- |
| *Roridula gorgonias* | Roridulaceae | CP | PP273159.1 | 175,274 | 43,851 | 131,423 |
| *Heliamphora minor* | Sarraceniaceae | CP | PP273160.1 | 160,156 | 34,468 | 125,688 |
| *Darlingtonia californica* | Sarraceniaceae | CP | PP273162.1 | 153,730 | 27,785 | 125,945 |
| *Sarracenia leucophylla* | Sarraceniaceae | CP | PP273161.1 | 155,159 | 27,692 | 127,467 |
| *Allotropa virgata* | Ericaceae | MP | NC_035580.1 | 35,983 | — | — |
| *Pityopus californicus* | Ericaceae | MP | NC_035584.1 | 35,926 | — | — |
| *Hemitomes congestum* | Ericaceae | MP | NC_035581.1 | 35,569 | — | — |
| *Monotropa uniflora* | Ericaceae | MP | NC_035582.1 | 43,351 | 8,422 | 34,929 |
| *Monotropsis odorata* | Ericaceae | MP | NC_035583.1 | 32,752 | — | — |
| *Mitrastemon kanehirai* | Mitrastemonaceae | HP | MF372930.1 | 25,740 | — | — |
| *Barringtonia racemosa* | Lecythidaceae | AP | NC_035705.1 | 159,002 | 26,058 | 132,944 |
| *Pterostyrax hispidus* | Styracaceae | AP | NC_041135.1 | 158,879 | 26,086 | 132,793 |
| *Saurauia tristyla* | Actinidiaceae | AP | NC_044098.1 | 156,676 | 23,960 | 132,716 |
| *Sladenia celastrifolia* | Sladeniaceae | AP | NC_035707.1 | 161,319 | 28,793 | 132,526 |
| *Diospyros hainanensis* | Ebenaceae | AP | NC_042160.1 | 157,999 | 25,822 | 132,177 |
| *Marcgravia coriacea* | Marcgraviaceae | AP | NC_041255.1 | 158,401 | 26,255 | 132,146 |
| *Fouquieria diguetii* | Fouquieriaceae | AP | MG524997.1 | 157,895 | 26,046 | 131,849 |
| *Pouteria campechiana* | Sapotaceae | AP | KX426215.1 | 157,922 | 26,120 | 131,802 |
| *Euryodendron excelsum* | Pentaphylacaceae | AP | NC_039178.1 | 157,702 | 26,072 | 131,630 |
| *Clethra fargesii* | Clethraceae | AP | NC_060326.1 | 157,486 | 25,980 | 131,506 |
| *Symplocos ovatilobata* | Symplocaceae | AP | NC_036489.1 | 157,417 | 26,089 | 131,328 |
| *Aegiceras corniculatum* | Primulaceae | AP | MN167882.1 | 157,241 | 25,970 | 131,271 |
| *Apterosperma oblata* | Theaceae | AP | NC_035641.1 | 156,590 | 26,082 | 130,508 |
| *Polemonium chinense* | Polemoniaceae | AP | NC_050355.1 | 155,578 | 25,735 | 129,843 |
| *Hydrocera triflora* | Balsaminaceae | AP | NC_037400.1 | 154,189 | 25,622 | 128,567 |

AP, CP, MP and HP denote obligate autotrophic, carnivorous, mycoheterotrophic and holoparasitic plants, respectively. IR and NR denote inverted repeat and non-redundant plastome retaining a single IR, respectively. "—" indicates absence of IR region.

**Table S2.** BLASTN hits of species-specific DNA regions of Ericales plastomes against the NCBI core nucleotide database (nt; database update: 24 December 2025), using BLASTN with the following parameters: word size = 11; match/mismatch = 2/−3; gap open = 5, gap extension = 2; *E*-value < 1e−5.

| **Insertions** | **Length** | **Location** | **BLASTN hit** | | | | | |
| --- | --- | --- | --- | --- | --- | --- | --- | --- |
|  | **(bp)** |  | **Coverage** | **Identity** | ***E*-value** | **Accession number** | **Species** | **Type** |
| Allotropa:7488-7554 | 67 | IG | — | — | — | — | — | — |
| Allotropa:7600-7637 | 38 | IG | — | — | — | — | — | — |
| Allotropa:11413-11629 | 217 | IG | — | — | — | — | — | — |
| Allotropa:11784-11901 | 118 | IG | 78% | 79.35% | 2E-09 | AC217038.1 | *Populus trichocarpa* | NG |
| Allotropa:12083-12109 | 27 | IG | — | — | — | — | *—* | — |
| Allotropa:12365-12479 | 115 | IG | — | — | — | — | *—* | — |
| Allotropa:12680-12743 | 64 | IG | — | — | — | — | *—* | — |
| Allotropa:12957-13039 | 83 | IG | — | — | — | — | *—* | — |
| Allotropa:13080-13210 | 131 | IG | 98% | 76.07% | 7E-09 | OZ373858.1 | *Paracoenia fumosa* | NG |
| Allotropa:14409-14436 | 28 | *rpl2* | — | — | — | — | *—* | — |
| Allotropa:14482-14565 | 84 | *rpl2* | — | — | — | — | *—* | — |
| Allotropa:16795-16908 | 114 | IG | 97% | 83.93% | 4E-17 | CR352226.16 | *Danio rerio* | NG |
| Allotropa:18572-18664 | 93 | IG | 68% | 82.54% | 2E-06 | OZ375659.1 | *Plasmodium malariae* | NG |
| Allotropa:27953-28022 | 70 | IG | 70% | 93.75% | 3E-09 | OZ312379.1 | *Benthosema glaciale* | NG |
| Allotropa:28091-28433 | 343 | IG | 99% | 68.45% | 5E-30 | OZ373322.1 | *Lotoria lotoria* | NG |
| Allotropa:30894-31001 | 108 | *accD* | — | — | — | — | *—* | — |
| Barringtonia:73987-74084 | 98 | *clpP* intron | 100% | 84.00% | 3E-17 | OR698943.1 | *Cypripedium yunnanense* | PT |
| Barringtonia:85561-85636 | 76 | *rpl16* intron | 88% | 86.76% | 9E-10 | FJ814682.1 | *Phacelia fremontii* | PT |
| Hemitomes:1650-1676 | 27 | IG | — | — | — | — | *—* | — |
| Hemitomes:4870-4972 | 103 | IG | 48% | 92.00% | 6E-08 | OZ335946.1 | *Phyllonorycter ulicicolella* | MT |
| Hemitomes:7211-7242 | 32 | IG | — | — | — | — | *—* | — |
| Hemitomes:7582-7656 | 75 | IG | — | — | — | — | *—* | — |
| Hemitomes:8156-8731 | 530 | IG | 59% | 74.60% | 4E-33 | OZ273963.1 | *Turdus pilaris* | NG |
| Hemitomes:9082-9176 | 95 | IG | — | — | — | — | *—* | — |
| Hemitomes:9300-9395 | 96 | IG | — | — | — | — | *—* | — |
| Hemitomes:13552-13579 | 28 | IG | — | — | — | — | *—* | — |
| Hemitomes:13879-13972 | 94 | IG | — | — | — | — | *—* | — |
| Hemitomes:19984-20091 | 108 | IG | 90% | 82.47% | 3E-13 | OZ373865.1 | *Paracoenia fumosa* | NG |
| Hemitomes:27345-27370 | 26 | IG | — | — | — | — | *—* | — |
| Hemitomes:33021-33052 | 32 | *rps2* | — | — | — | — | *—* | — |
| Hemitomes:33105-33268 | 164 | *rps2* | 96% | 75.00% | 5E-12 | OZ311877.2 | *Steromphala pennanti* | NG |
| Mitrastemon:1060-1109 | 50 | *rps2* | — | — | — | — | *—* | — |
| Mitrastemon:1249-1311 | 63 | *rps2* | — | — | — | — | *—* | — |
| Mitrastemon:1343-1371 | 29 | *rps2* | — | — | — | — | *—* | — |
| Mitrastemon:1987-2294 | 308 | *accD* | 75% | 72.57% | 2E-16 | LT996319.1 | *Philodendron sonderianum* | PT |
| Mitrastemon:2339-2501 | 163 | *accD* | 65% | 84.91% | 8E-23 | XM_009019577.1 | *Helobdella robusta* | NG |
| Mitrastemon:4599-4710 | 112 | *clpP* intron | 63% | 84.51% | 6E-09 | AP038991.1 | *Polyrhachis lamellidens* | NG |
| Mitrastemon:4785-5035 | 251 | *clpP* intron | 45% | 78.26% | 3E-12 | BX004996.9 | *Danio rerio* | NG |
| Mitrastemon:5335-5430 | 96 | *clpP* intron | 80% | 81.01% | 8E-06 | NC_070358.1 | *Monocostus uniflorus* | PT |
| Mitrastemon:5453-5628 | 176 | *clpP* intron | 66% | 80.00% | 3E-16 | OE840010.1 | *Timema genevievae* | NG |
| Mitrastemon:5677-5710 | 34 | *clpP* intron | — | — | — | — | *—* | — |
| Mitrastemon:5795-5836 | 42 | IG | — | — | — | — | *—* | — |
| Mitrastemon:6737-6786 | 50 | IG | 100% | 89.80% | 2E-07 | PP764102.1 | *Drosophila melanogaster* | MT |
| Mitrastemon:6977-7093 | 117 | *rps8* | — | — | — | — | *—* | — |
| Mitrastemon:7165-7302 | 138 | *rpl16* | 79% | 78.18% | 4E-12 | PQ580725.1 | *Malus asiatica* | PT |
| Mitrastemon:7625-7663 | 39 | *rpl16* | — | — | — | — | *—* | — |
| Mitrastemon:9201-9274 | 74 | *rpl2* intron | — | — | — | — | *—* | — |
| Mitrastemon:9291-9459 | 169 | *rpl2* intron | 84% | 74.31% | 2E-10 | OR698930.1 | *Cypripedium flavum* | NG |
| Mitrastemon:10391-10442 | 52 | IG | 75% | 94.74% | 8E-06 | KT206599.1 | *Saccharomyces cerevisiae* | NG |
| Mitrastemon:12644-12669 | 26 | IG | — | — | — | — | *—* | — |
| Mitrastemon:12926-12982 | 57 | *rrn23* | — | — | — | — | *—* | — |
| Mitrastemon:13027-13087 | 61 | *rrn23* | 100% | 89.66% | 7E-09 | OZ377182.1 | *Plagiochila carringtonii* | NG |
| Mitrastemon:14237-14323 | 87 | *rrn23* | — | — | — | — | *—* | — |
| Mitrastemon:15991-16055 | 65 | *ycf1* | 88% | 91.07% | 5E-11 | PQ142143.1 | *Apis cerana* | NG |
| Mitrastemon:16192-16225 | 34 | *ycf1* | — | — | — | — | *—* | — |
| Mitrastemon:16338-16545 | 208 | *ycf1* | 32% | 86.57% | 1E-08 | MW279214.1 | *Psyttalia lounsburyi* | MT |
| Mitrastemon:16579-16791 | 213 | *ycf1* | 100% | 74.05% | 4E-15 | OZ373859.1 | *Paracoenia fumosa* | NG |
| Mitrastemon:16992-17204 | 213 | *ycf1* | 100% | 76.30% | 8E-18 | LR862152.1 | *Ananas comosus var. bracteatus* | NG |
| Mitrastemon:17255-17767 | 513 | *ycf1* | 88% | 75.45% | 2E-63 | OZ276500.1 | *Flustra foliacea* | NG |
| Mitrastemon:18225-18340 | 116 | *ycf1* | 100% | 76.92% | 2E-08 | OZ373858.1 | *Paracoenia fumosa* | NG |
| Mitrastemon:18502-18682 | 181 | *ycf1* | — | — | — | — | *—* | — |
| Mitrastemon:18714-18760 | 47 | *ycf1* | 96% | 91.11% | 5E-06 | OZ373324.1 | *Lotoria lotoria* | NG |
| Mitrastemon:18785-18959 | 175 | *ycf1* | 61% | 79.09% | 2E-11 | CP099823.1 | *Candidatus Shikimatogenerans bostrichidophilus* | NG |
| Mitrastemon:19035-19222 | 188 | *ycf1* | 52% | 81.00% | 2E-11 | XM_055522355.1 | *Condylostylus longicornis* | NG |
| Mitrastemon:19468-19493 | 26 | *ycf1* | — | — | — | — | *—* | — |
| Mitrastemon:19601-19697 | 97 | *ycf1* | 78% | 78.67% | 8E-06 | OZ375492.1 | *Clubiona pallidula* | NG |
| Mitrastemon:19770-19857 | 88 | *ycf1* | 84% | 80.26% | 2E-06 | CP159812.1 | *Candidatus Pelagibacter sp. Uisw_136* | NG |
| Mitrastemon:19875-20045 | 171 | *ycf1* | 96% | 72.78% | 6E-12 | CP158689.2 | *Candidatus Shikimatogenerans sp. Ttur* | NG |
| Mitrastemon:20184-20294 | 111 | *ycf1* | 67% | 79.75% | 9E-07 | OZ376761.1 | *Clinocardium nuttallii* | NG |
| Mitrastemon:20825-20859 | 35 | *ycf2* | — | — | — | — | *—* | — |
| Mitrastemon:20892-20946 | 55 | *ycf2* | — | — | — | — | *—* | — |
| Mitrastemon:20983-21304 | 322 | *ycf2* | 74% | 77.97% | 4E-12 | OZ373859.1 | *Paracoenia fumosa* | NG |
| Mitrastemon:21353-21550 | 198 | *ycf2* | 94% | 75.18% | 2E-13 | OZ364090.2 | *Euprymna scolopes* | NG |
| Mitrastemon:21592-21647 | 56 | *ycf2* | — | — | — | — | *—* | — |
| Mitrastemon:21806-21947 | 142 | *ycf2* | — | — | — | — | *—* | — |
| Mitrastemon:22358-22539 | 182 | *ycf2* | 56% | 78.85% | 1E-08 | OZ373858.1 | *Paracoenia fumosa* | NG |
| Mitrastemon:22684-22730 | 47 | *ycf2* | — | — | — | — | *—* | — |
| Mitrastemon:22951-23054 | 104 | *ycf2* | — | — | — | — | *—* | — |
| Mitrastemon:23137-24221 | 1085 | *ycf2* | 99% | 67.68% | 1E-43 | PP941955.1 | *Speranskia tuberculata* | PT |
| Mitrastemon:24264-25112 | 849 | *ycf2* | 100% | 68.49% | 5E-35 | OR698939.1 | *Cypripedium plectrochilum* | PT |
| Mitrastemon:25183-25238 | 56 | *ycf2* | — | — | — | — | *—* | — |
| Mitrastemon:25283-25529 | 247 | *ycf2* | 48% | 78.99% | 1E-10 | CP144848.1 | *Candidatus Stammera capleta* | NG |
| Monotropa:7406-7435 | 30 | IG | 79% | 100.00% | 9E-02 | OY979795.1 | *Helicoverpa assulta* | NG |
| Monotropa:7467-7497 | 31 | IG | — | — | — | — | *—* | — |
| Monotropa:7843-7887 | 45 | IG | — | — | — | — | *—* | — |
| Monotropa:7925-7966 | 42 | IG | — | — | — | — | *—* | — |
| Monotropa:8222-8301 | 80 | IG | — | — | — | — | *—* | — |
| Monotropa:10725-10792 | 68 | IG | — | — | — | — | *—* | — |
| Monotropa:13707-14018 | 312 | IG | 30% | 77.17% | 3E-06 | OZ373865.1 | *Paracoenia fumosa* | NG |
| Monotropa:14258-14292 | 35 | IG | — | — | — | — | *—* | — |
| Monotropa:14321-14347 | 27 | IG | — | — | — | — | *—* | — |
| Monotropa:15053-15316 | 264 | IG | 61% | 74.55% | 2E-13 | MW367981.1 | *Saccharomyces cerevisiae x Saccharomyces paradoxus* | MT |
| Monotropa:16061-16343 | 283 | IG | 95% | 77.78% | 3E-19 | OZ373858.1 | *Paracoenia fumosa* | NG |
| Monotropa:16610-16846 | 237 | IG | 49% | 75.65% | 2E-07 | LR031875.1 | *Brassica oleracea* | NG |
| Monotropa:18389-18472 | 84 | *accD* | — | — | — | — | *—* | — |
| Monotropa:18502-18936 | 435 | *accD* | 20% | 81.58% | 9E-09 | OZ374981.1 | *Limacia clavigera* | NG |
| Monotropa:18964-19033 | 70 | *accD* | — | — | — | — | *—* | — |
| Monotropa:19072-19458 | 387 | *accD* | 28% | 78.38% | 4E-13 | XM_028684188.1 | *Plasmodium sp. gorilla clade G2* | NG |
| Monotropa:19560-19656 | 97 | *accD* | — | — | — | — | *—* | — |
| Monotropa:19730-19820 | 91 | *accD* | — | — | — | — | *—* | — |
| Monotropa:21701-21923 | 223 | IG | 70% | 75.17% | 3E-17 | OZ244916.1 | *Gammaproteobacteria bacterium* | NG |
| Monotropa:22028-22121 | 94 | IG | — | — | — | — | *—* | — |
| Monotropa:22501-22617 | 117 | IG | — | — | — | — | *—* | — |
| Monotropa:36794-36937 | 144 | *accD* | 94% | 75.56% | 5E-12 | OZ374350.1 | *Eulalia xanthomucosa* | NG |
| Monotropa:36971-37296 | 326 | *accD* | 85% | 72.31% | 1E-18 | AP042721.1 | *Ciona intestinalis* | NG |
| Monotropa:37316-37465 | 150 | *accD* | — | — | — | — | *—* | — |
| Monotropa:37544-37820 | 277 | *accD* | — | — | — | — | *—* | — |
| Monotropa:37864-38097 | 234 | *accD* | — | — | — | — | *—* | — |
| Monotropa:38150-38191 | 42 | *accD* | — | — | — | — | *—* | — |
| Monotropa:38336-38541 | 206 | *accD* | — | — | — | — | *—* | — |
| Monotropa:38585-38703 | 119 | *accD* | — | — | — | — | *—* | — |
| Monotropa:38729-38861 | 133 | *accD* | — | — | — | — | *—* | — |
| Monotropa:38917-38949 | 33 | *accD* | — | — | — | — | *—* | — |
| Monotropa:38977-39042 | 66 | *accD* | — | — | — | — | *—* | — |
| Monotropa:39310-39560 | 251 | *accD* | 33% | 79.52% | 9E-06 | OZ373859.1 | *Paracoenia fumosa* | NG |
| Monotropa:3489-3643 | 155 | IG | — | — | — | — | *—* | — |
| Monotropa:3897-3984 | 88 | IG | 47% | 97.56% | 5E-08 | CP099827.1 | *Candidatus Shikimatogenerans bostrichidophilus* | NG |
| Monotropa:4026-4169 | 144 | IG | — | — | — | — | *—* | — |
| Monotropa:4262-4342 | 81 | IG | — | — | — | — | *—* | — |
| Monotropsis:3502-3583 | 82 | IG | — | — | — | — | *—* | — |
| Monotropsis:3745-3811 | 67 | IG | — | — | — | — | *—* | — |
| Monotropsis:3841-4010 | 170 | IG | 35% | 96.23% | 6E-12 | OZ251419.1 | *Gobiusculus flavescens* | NG |
| Monotropsis:4105-4949 | 845 | IG | 87% | 72.41% | 8E-70 | OZ376112.1 | *Agnoea josephinae* | NG |
| Monotropsis:6818-6883 | 66 | IG | — | — | — | — | *—* | — |
| Monotropsis:6940-7035 | 96 | IG | — | — | — | — | *—* | — |
| Monotropsis:7091-7291 | 201 | IG | — | — | — | — | *—* | — |
| Monotropsis:7512-7627 | 116 | IG | 77% | 84.88% | 1E-12 | OZ377387.1 | *Codakia orbicularis* | NG |
| Monotropsis:7679-7772 | 94 | IG | — | — | — | — | *—* | — |
| Monotropsis:7799-7991 | 193 | IG | — | — | — | — | *—* | — |
| Monotropsis:8032-8556 | 525 | IG | 13% | 84.06% | 1E-08 | XM_062080984.1 | *Apis cerana* | NG |
| Monotropsis:8589-8634 | 46 | IG | — | — | — | — | *—* | — |
| Monotropsis:11768-11831 | 64 | IG | — | — | — | — | *—* | — |
| Monotropsis:12033-12066 | 34 | IG | — | — | — | — | *—* | — |
| Monotropsis:12141-12192 | 52 | IG | — | — | — | — | *—* | — |
| Monotropsis:12396-12471 | 76 | IG | — | — | — | — | *—* | — |
| Monotropsis:12994-13035 | 42 | IG | — | — | — | — | *—* | — |
| Monotropsis:19888-19913 | 26 | IG | — | — | — | — | *—* | — |
| Monotropsis:20439-20477 | 39 | IG | — | — | — | — | *—* | — |
| Monotropsis:20501-20526 | 26 | *rps18* | — | — | — | — | *—* | — |
| Monotropsis:21495-21545 | 51 | IG | — | — | — | — | *—* | — |
| Monotropsis:21675-21714 | 40 | IG | — | — | — | — | *—* | — |
| Monotropsis:21812-21845 | 34 | IG | — | — | — | — | *—* | — |
| Monotropsis:21886-22173 | 288 | IG | 24% | 84.06% | 6E-09 | XM_062080984.1 | *Apis cerana* | NG |
| Monotropsis:22237-22341 | 105 | IG | — | — | — | — | *—* | — |
| Monotropsis:22374-22419 | 46 | IG | — | — | — | — | *—* | — |
| Monotropsis:22913-23155 | 243 | IG | 42% | 80.00% | 4E-10 | NC_069880.1 | *Paphiopedilum lowii* | PT |
| Monotropsis:23180-23345 | 166 | IG | — | — | — | — | *—* | — |
| Monotropsis:23390-23510 | 121 | IG | — | — | — | — | *—* | — |
| Monotropsis:23567-23599 | 33 | IG | — | — | — | — | *—* | — |
| Monotropsis:23631-23679 | 49 | IG | — | — | — | — | *—* | — |
| Monotropsis:23712-23875 | 164 | IG | — | — | — | — | *—* | — |
| Monotropsis:23944-23971 | 28 | IG | — | — | — | — | *—* | — |
| Monotropsis:28211-28249 | 39 | *accD* | — | — | — | — | *—* | — |
| Monotropsis:30544-30577 | 34 | *rps2* | — | — | — | — | *—* | — |
| Pityopus:7627-7676 | 50 | IG | — | — | — | — | *—* | — |
| Pityopus:12353-12399 | 47 | IG | — | — | — | — | *—* | — |
| Pityopus:14631-14656 | 26 | IG | — | — | — | — | *—* | — |
| Pityopus:31092-31119 | 28 | *accD* | — | — | — | — | *—* | — |
| Pityopus:31265-31428 | 164 | *accD* | 99% | 76.40% | 1E-20 | XM_020040489.3 | *Esox lucius* | NG |
| Pityopus:35312-35376 | 65 | IG | — | — | — | — | *—* | — |
| Roridula:71471-71555 | 85 | IG | — | — | — | — | *—* | — |
| Roridula:71684-71756 | 73 | IG | — | — | — | — | *—* | — |
| Roridula:72010-72405 | 396 | IG | 36% | 71.33% | 3E-08 | OZ373250.1 | *Carcinus aestuarii* | NG |
| Roridula:84675-84735 | 61 | IG | — | — | — | — | *—* | — |
| Roridula:87142-87300 | 159 | *ycf2* | — | — | — | — | *—* | — |
| Roridula:90087-90306 | 220 | *ycf2* | 99% | 78.50% | 2E-39 | OZ375011.1 | *Meledella werneri* | NG |
| Roridula:90830-91020 | 191 | *ycf2* | 32% | 85.25% | 1E-07 | OZ374238.1 | *Erebia pandrose* | NG |
| Roridula:91665-91801 | 137 | *ycf2* | — | — | — | — | *—* | — |
| Roridula:92923-93077 | 155 | *ycf2* | — | — | — | — | *—* | — |
| Roridula:93173-93286 | 114 | *ycf2* | — | — | — | — | *—* | — |
| Roridula:93423-93630 | 208 | *ycf2* | 44% | 84.78% | 3E-17 | OZ375005.1 | *Meledella werneri* | NG |
| Roridula:110103-110205 | 103 | *ycf1* | — | — | — | — | *—* | — |
| Roridula:110418-110459 | 42 | *ycf1* | — | — | — | — | *—* | — |
| Roridula:110807-110857 | 51 | *ycf1* | — | — | — | — | *—* | — |
| Roridula:111467-111512 | 46 | *ycf1* | — | — | — | — | *—* | — |
| Roridula:111541-111719 | 179 | *ycf1* | — | — | — | — | *—* | — |
| Roridula:111740-112052 | 313 | *ycf1* | — | — | — | — | *—* | — |
| Roridula:112128-112527 | 400 | *ycf1* | 21% | 87.14% | 5E-11 | OZ295850.1 | *Dracocephalum ruyschiana* | NG |
| Roridula:112713-112763 | 51 | *ycf1* | — | — | — | — | *—* | — |
| Roridula:112902-113222 | 321 | *ycf1* | 43% | 75.54% | 2E-14 | NC_059001.1 | *Diospyros nigra* | PT |
| Roridula:114903-116453 | 1551 | *ycf1* | 75% | 78.10% | 0E+00 | XM_040438304.1 | *Bufo bufo* | NG |
| Roridula:116481-117119 | 639 | *ycf1* | 92% | 74.95% | 2E-76 | OZ364081.2 | *Euprymna scolopes* | NG |
| Roridula:117178-117403 | 226 | *ycf1* | — | — | — | — | *—* | — |
| Saurauia:73352-73600 | 249 | *clpP* intron | — | — | — | — | *—* | — |
| Saurauia:73622-73843 | 222 | *clpP* intron | 26% | 92.59% | 1E-10 | OZ364084.2 | *Euprymna scolopes* | NG |

The numbers before and after the connecting line in the first column represent the starting and ending positions of insertions on the corresponding plastome. IG: intergenic regions; NG: nuclear genome; PG: plastid genome; MG: mitochondrial genome.

**Table S3.** Plastid RNA editing sites of RS carnivorous plants and two autotrophic model plants

| **Editing Site** | **Base** | | | | | | **AA Postion** | **Codon Position** | **Codon Change** | **Amino Change** |
| --- | --- | --- | --- | --- | --- | --- | --- | --- | --- | --- |
|  | **Dar** | **Hel** | **Sar** | **Ror** | **Nic** | **Sol** |  |  |  |  |
| *ndhA*-341 | C | - | - | C | C | C | 114 | 2 | UCA->UUA | Ser -> Leu |
| *ndhA*-1073 | - | - | - | C | C | C | 358 | 2 | UCC->UUC | Ser -> Phe |
| *ndhB*-149 | C | C | C | C | C | C | 50 | 2 | UCA->UUA | Ser -> Leu |
| *ndhB*-467 | C | C | C | C | C | C | 156 | 2 | CCA->CUA | Pro -> Leu |
| *ndhB*-586 | C | C | C | C | C | C | 196 | 1 | CAU->UAU | His -> Tyr |
| *ndhB*-611 | C | C | C | C | C | C | 204 | 2 | UCA->UUA | Ser -> Leu |
| *ndhB*-737 | C | C | C | C | C | C | 246 | 2 | CCA->CUA | Pro -> Leu |
| *ndhB*-746 | C | C | C | C | C | C | 249 | 2 | UCU->UUU | Ser -> Phe |
| *ndhB*-830 | C | C | C | C | C | C | 277 | 2 | UCA->UUA | Ser -> Leu |
| *ndhB*-836 | T | T | T | T | C | C | 279 | 2 | UCA->UUA | Ser -> Leu |
| *ndhB*-1481 | C | C | C | C | C | C | 494 | 2 | CCA->CUA | Pro -> Leu |
| *ndhC*-40 | C | T | T | C | C | C | 14 | 1 | CUA->UUA | Leu -> Leu |
| *ndhD*-2 | - | C | C | C | C | C | 1 | 2 | ACG->AUG | Thr -> Met |
| *ndhD*-383 | T | T | T | C | C | T | 128 | 2 | UCA->UUA | Ser -> Leu |
| *ndhD*-599 | T | T | T | T | C | C | 200 | 2 | UCA->UUA | Ser -> Leu |
| *ndhD*-674 | T | T | T | T | C | C | 225 | 2 | UCG->UUG | Silence |
| *ndhD*-878 | C | C | C | C | T | C | 293 | 2 | UCA->UUA | Ser -> Leu |
| *ndhD*-1298 | - | T | T | T | C | C | 433 | 2 | UCA->UUA | Ser -> Leu |
| *ndhD*-1310 | - | T | T | C | C | C | 437 | 2 | UCA->UUA (Sol: CCA->CUA) | Ser -> Leu (Sol: Pro -> Leu) |
| *ndhF*-290 | - | - | C | C | C | C | 97 | 2 | UCA->UUA | Ser -> Leu |
| *ndhG*-50 | - | - | T | T | C | C | 17 | 2 | UCG->UUG | Silence |
| *ndhG*-347 | - | T | T | T | C | C | 116 | 2 | UCA->UUA | Ser -> Leu |
| *accD*-1460 | C | C | C | C | T | T | 487 | 2 | CCU -> CUU | Pro -> Leu |
| *atpA*-791 | C | C | C | C | C | T | 264 | 2 | CCC->CUC (Sar/Hel: CCU -> CUU) | Pro -> Leu |
| *atpA*-795 | C | C | C | C | C | C | 265 | 3 | UCC->UCU | Silence |
| *atpA*-914 | C | C | C | T | T | T | 305 | 2 | UCA -> UUA | Ser -> Leu |
| *atpB*-828 | C | C | C | C | C | C | 276 | 3 | UUC -> UUU | Silence |
| *atpF*-92 | C | C | T | C | C | C | 31 | 2 | CCA->CUA | Pro -> Leu |
| *matK*-161 | T | C | C | T | T | T | 54 | 2 | UCC -> UUC | Ser -> Phe |
| *matK*-264 | C | C | C | C | C | C | 88 | 3 | AAC -> AAU | Silence |
| *matK*-469 | C | C | C | C | T | T | 157 | 1 | CAC -> UAC | His -> Tyr |
| *matK*-725 | C | C | C | C | T | T | 242 | 2 | UCU -> UUU | Ser -> Phe |
| *matK*-1258 | C | T | C | C | T | T | 420 | 1 | CAU -> UAU | His -> Tyr |
| *petB*-12 | C | C | C | C | A | A | 4 | 3 | GUC -> GUU | Silence |
| *petB*-611 | C | C | C | C | C | C | 204 | 2 | CCA->CUA | Pro -> Leu |
| *petL*-5 | C | C | C | C | T | T | 2 | 2 | CCU->CUU | Pro -> Leu |
| *psaI*-80 | C | C | C | C | T | T | 27 | 2 | UCU -> UUU | Ser -> Phe |
| *psaI*-85 | C | C | C | C | C | C | 29 | 1 | CAU -> UAU | His -> Tyr |
| *psbB*-414 | C | C | C | C | C | C | 138 | 3 | AUC -> AUU | Silence |
| *psbE*-214 | C | C | C | C | C | C | 72 | 1 | CCU->UCU | Pro -> Ser |
| *psbF*-77 | C | C | C | C | T | T | 26 | 2 | UCU->UUU | Ser -> Phe |
| *psbL*-2 | T | T | T | T | C | C | 1 | 2 | ACG->AUG | Thr -> Met |
| *psbL*-111 | C | C | C | C | C | C | 37 | 3 | UUC -> UUU | Silence |
| *psbZ*-50 | C | C | C | C | T | T | 17 | 2 | UCA->UUA | Ser -> Leu |
| *rpl16*-12 | C | C | C | C | C | C | 4 | 3 | CCC -> CCU | Pro -> Pro |
| *rpl20*-308 | T | T | T | T | C | C | 103 | 2 | UCA->UUA | Ser -> Leu |
| *rpl23*-71 | C | C | C | T | C | C | 24 | 2 | UCU->UUU | Ser -> Phe |
| *rpl23*-89 | C | C | C | C | C | C | 30 | 2 | UCA->UUA | Ser -> Leu |
| *rpoA*-65 | C | C | C | A | C | C | 22 | 2 | ACA->AUA | Thr -> Ile |
| *rpoA*-200 | C | C | C | T | C | C | 67 | 2 | UCU->UUU | Ser -> Phe |
| *rpoA*-368 | C | C | C | T | T | T | 123 | 2 | UCA -> UUA | Ser -> Leu |
| *rpoA*-830 | T | T | T | T | C | C | 277 | 2 | UCA->UUA | Ser -> Leu |
| *rpoB*-338 | C | C | C | C | C | C | 113 | 2 | UCU->UUU | Ser -> Phe |
| *rpoB*-473 | C | C | C | C | C | C | 158 | 2 | UCA->UUA | Ser -> Leu |
| *rpoB*-551 | C | C | C | C | C | C | 184 | 2 | UCA->UUA | Ser -> Leu |
| *rpoB*-566 | C | C | C | C | T | T | 189 | 2 | UCG -> UUG | Silence |
| *rpoB*-1382 | C | C | C | C | C | C | 461 | 2 | UCA -> UUA | Ser -> Leu |
| *rpoB*-2000 | C | C | C | C | C | C | 667 | 2 | UCU->UUU | Ser -> Phe |
| *rpoB*-2426 | T | T | T | T | T | C | 809 | 2 | UCA->UUA | Ser -> Leu |
| *rpoC1*-62 | C | C | C | C | C | C | 21 | 2 | UCA->UUA | Ser -> Leu |
| *rpoC1*-509 | C | C | C | C | T | T | 170 | 2 | UCA->UUA | Ser -> Leu |
| *rpoC1*-1868 | C | C | C | T | C | C | 623 | 2 | UCA -> UUA | Ser -> Leu |
| *rpoC2*-2782 | C | C | C | C | C | C | 928 | 1 | CAA -> UAA | Gln -> * |
| *rpoC2*-2864 | C | C | C | C | C | C | 955 | 2 | UCU -> UUU | Ser -> Phe |
| *rpoC2*-3731 | C | C | C | C | C | C | 1244 | 2 | UCA->UUA (Dar: UCG -> UUG) | Ser -> Leu (Dar: Silence) |
| *rps2*-134 | C | C | C | C | C | C | 45 | 2 | ACA->AUA | Thr -> Ile |
| *rps2*-248 | C | C | C | C | C | C | 83 | 2 | UCA->UUA | Ser -> Leu |
| *rps8*-182 | C | C | C | C | T | T | 61 | 2 | UCA -> UUA | Ser -> Leu |
| *rps11*-108 | T | C | C | C | C | C | 36 | 3 | UUC -> UUU | Silence |
| *rps12*-221 | C | C | C | C | T | C | 74 | 2 | UCA->UUA | Ser -> Leu |
| *rps14*-80 | C | C | C | C | C | C | 27 | 2 | UCA->UUA | Ser -> Leu |
| *rps14*-149 | C | C | C | T | C | T | 50 | 2 | CCA->CUA | Pro -> Leu |
| *rps18*-242 | C | C | C | C | T | T | 81 | 2 | UCG -> UUG | Silence |
| *clpP* intron 1-176 | C | C | C | - | C | C | ○ | ○ | ○ | ○ |
| *clpP* intron 1-426 | C | C | A | - | C | C | ○ | ○ | ○ | ○ |
| *clpP* intron 2-309 | C | C | C | - | C | C | ○ | ○ | ○ | ○ |
| *clpP* intron 2-414 | C | C | C | - | C | C | ○ | ○ | ○ | ○ |
| *petD* intron 1-690 (Sar) | G | G | C | A | - | - | ○ | ○ | ○ | ○ |
| *rps12* intron 1-186 | C | C | C | C | T | T | ○ | ○ | ○ | ○ |
| *rps12* intron 1-327 | C | A | A | C | C | C | ○ | ○ | ○ | ○ |
| *rps16* intron 1-439 | T | T | T | C | C | C | ○ | ○ | ○ | ○ |
| *ycf3* intron 2-355 | C | C | C | C | C | C | ○ | ○ | ○ | ○ |

Dar: *Darlingtonia californica*; Hel: *Heliamphora minor*; Sar: *Sarracenia leucophylla*; Ror: *Roridula gorgonias*; Nic: *Nicotiana tabacum*; Sol: *Solanum lycopersicum*. The naming of RNA editing sites indicates the name of the region where the editing site is located and the base position. The base position is standardized based on the plastid genes of *Nicotiana tabacum*. In cases where the site is not present in *Nicotiana tabacum*, the plastid genes of *Sarracenia leucophylla* are utilized to determine the base position. The cross symbol represents a pseudogene site, while "-" denotes a deleted site. The bright yellow filling indicates the detected RNA editing site (editing efficiency > 10%, maximum variant *P*-value < 10^-6^).
